# Photoswitchable Diazocine Derivative for Adenosine A_3_ Receptor Activation in Psoriasis

**DOI:** 10.1021/jacs.4c13558

**Published:** 2024-12-16

**Authors:** Marc López-Cano, Mirko Scortichini, Dilip K. Tosh, Veronica Salmaso, Tongil Ko, Glòria Salort, Ingrid Filgaira, Concepció Soler, Dirk Trauner, Jordi Hernando, Kenneth A. Jacobson, Francisco Ciruela

**Affiliations:** † Pharmacology Unit, Department of Pathology and Experimental Therapeutics, Faculty of Medicine and Health Sciences, Institute of Neurosciences, University of Barcelona, L′Hospitalet de Llobregat 08907, Spain; ‡ Neuropharmacology and Pain Group, Neuroscience Program, Bellvitge Biomedical Research Institute, L′Hospitalet de Llobregat 08907, Spain; § Molecular Recognition Section, Laboratory of Bioorganic Chemistry, NIDDK, National Institutes of Health, Bethesda, Maryland 20892, United States; ∥ Department of Chemistry, University of Pennsylvania College of Arts and Sciences, Philadelphia, Pennsylvania 19104, United States; ⊥ Immunology Unit, Department of Pathology and Experimental Therapeutics, Faculty of Medicine and Health Sciences, University of Barcelona, L′Hospitalet de Llobregat 08907, Spain; # Immunity, Inflammation and Cancer Group, Oncology Program, Bellvitge Biomedical Research Institute, L′Hospitalet de Llobregat 08907, Spain; ∇ Department of Chemistry, New York University, New York City, New York 10003, United States; ○ Department of Chemistry, Autonomous University of Barcelona, Cerdanyola del Vallès 08193, Spain

## Abstract

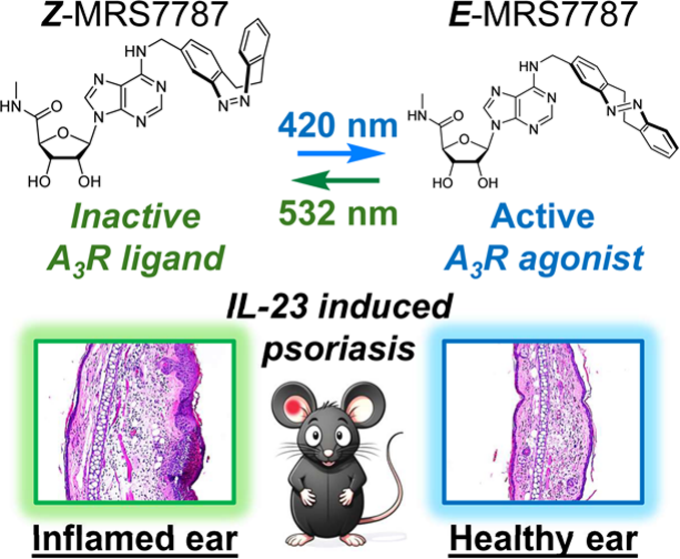

Incorporating photoisomerizable moieties within drugs offers the possibility of rapid and reversible light-dependent switching between active and inactive configurations. Here, we developed a photoswitchable adenosine A_3_ receptor (A_3_R) agonist that confers optical control on this G protein-coupled receptor through noninvasive topical skin irradiation in an animal model of psoriasis. This was achieved by covalently bonding an adenosine-5′-methyluronamide moiety to a diazocine photochrome, whose singular photoswitching properties facilitated repeated interconversion between a thermally stable, biologically inactive *Z* agonist form and a photoinduced, pharmacologically active *E* configuration. As a result, our photoswitchable agonist allowed the precise modulation of A_3_R function both *in vitro* and *in vivo*, which led to a clear light-controlled pharmacotherapeutic effect on mouse skin lesions. This breakthrough not only demonstrates the potential of diazocine photoswitches for *in vivo* photopharmacology but also paves the way for the development of new strategies for skin-related diseases that require localized and temporally controlled drug action.

## Introduction

Photopharmacology uses light to convert an inactive drug into its biologically active form at the specific site of action within the body. This can be achieved by altering the molecular configuration of the drug through a process known as photoswitching or by unmasking its activity *via* photouncaging.^1^ Photoswitching involves the reversible conversion of a drug between different structural states upon exposure to light, allowing its biological activity to be switched on and off with high precision. On the other hand, photouncaging refers to the release of a previously masked, inactive drug by the application of light, which removes the protective group, often a coumarin derivative, thus restoring the original drug’s full pharmacological capacity. Both types of photodrugs offer the potential for precise control over when and where a drug becomes active, improve therapeutic specificity, and reduce off-target effects. As the field matures, scientists hope to develop more advanced molecules to apply photopharmacology to living systems, especially in a myriad of specific therapeutic areas, including cancer, neurological disorders, infectious diseases, and inflammatory-related disorders.

Azobenzene-based photoswitchable drugs are often preferred over photocaged ligands because they can reversibly switch between active and inactive configurations through a photoisomerization process, thus providing the ability to modify the intrinsic activity of the drug on and off in response to light without generating any byproducts (*i.e.*, coumarin).^1^ However, azobenzene-based photodrugs often exhibit an undesirable pharmacological profile for *in vivo* therapeutic use: they are typically active in their thermally stable form in the dark, they become inactive (or less active) after exposure to light.^2^ Interestingly, diazocines, a class of bridged azobenzenes, are unique in that they photoconvert between a dark-adapted ***Z*** isomer and a photoinduced ***E*** form,^3^ thus overcoming the potential pharmacotherapeutic limitations of conventional azobenzene photodrug,^2,4,5^ offering a more reliable way to control drug activity with light. Despite some successful examples of diazocine-based photoswitchable drugs demonstrated *in vitro*,^2,4−8^ their application in living organisms has yet to be demonstrated. Here, we tackle this challenge by introducing a diazocine derivative as a photodrug that targets the adenosine A_3_ receptor (A_3_R) in an animal model of psoriasis.

**Figure 1:**
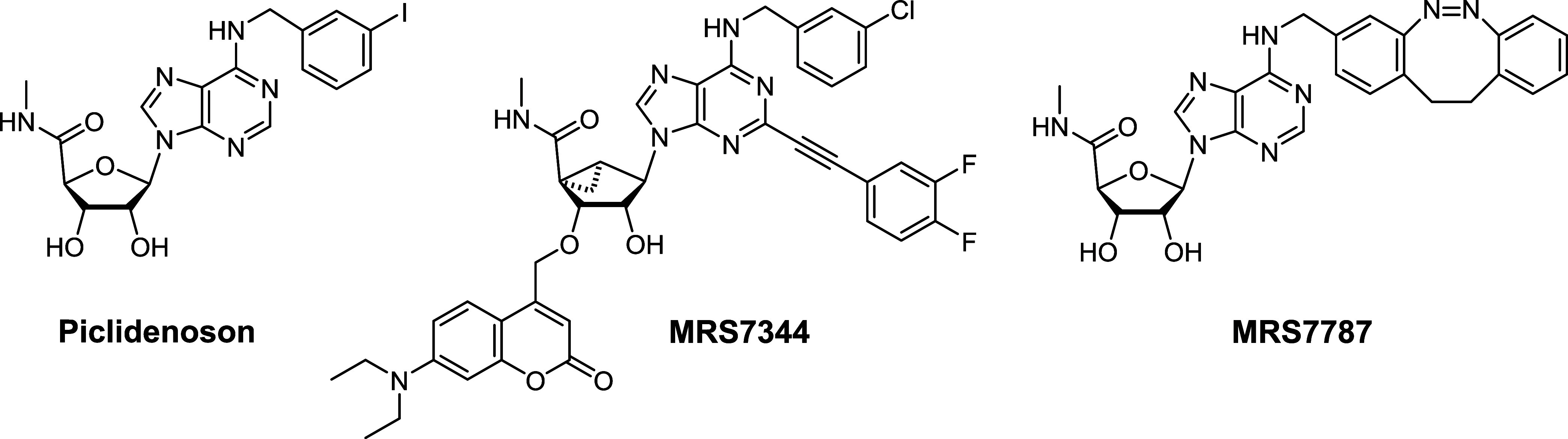
Chemical structures of Piclidenoson, MRS7344, and MRS7787.

Adenosine exerts its effects through four G protein-coupled receptor (GPCR) subtypes, A_1_R, A_2A_R, A_2B_R, and A_3_R. These receptors are of significant interest in the treatment of chronic diseases, such as inflammation, as they mediate various pathways that regulate immune responses.^9^ In recent years, A_3_R, a Gi guanine nucleotide binding protein-coupled receptor, has been identified as a key player in a variety of primary cells involved in inflammatory responses,^10^ making it a promising target for the treatment of certain inflammatory conditions. Psoriasis, a chronic inflammatory condition, involves both skin and systemic symptoms. Although current treatments have demonstrated excellent clinical efficacy in many patients, they are not curative and often prove inadequate in others. Topical therapies are commonly used for low to mild-severity cases or localized skin lesions, while systemic drug treatments are typically prescribed for more severe or widespread conditions. Despite the available treatment options, there is a clear need for new and improved therapies to address some of the limitations of existing approaches. In this context, new orally active drugs are being developed for the management of moderate to severe psoriasis,^11^ inclusing Piclidenoson Figure 1, an A_3_R agonist. Although the development of selective and potent A_3_R agonists has great potential for the treatment of psoriasis and other inflammatory conditions, the widespread expression of A_3_R within the organism can eventually lead to side effects.^12−15^ Hence, local, light-mediated activation of A_3_R in psoriatic lesions could minimize systemic exposure and reduce the risk of off-target effects. In this regard, other A_3_R photodrug options (*i.e.*, MRS7344; Figure 1) have been proposed.^16^ However, MRS7344 is a photocaged drug that is inactive while kept in the dark as the masked form and activated when light is illuminated. Unfortunately, this process is necessarily an irreversible transformation.

**Figure 2:**
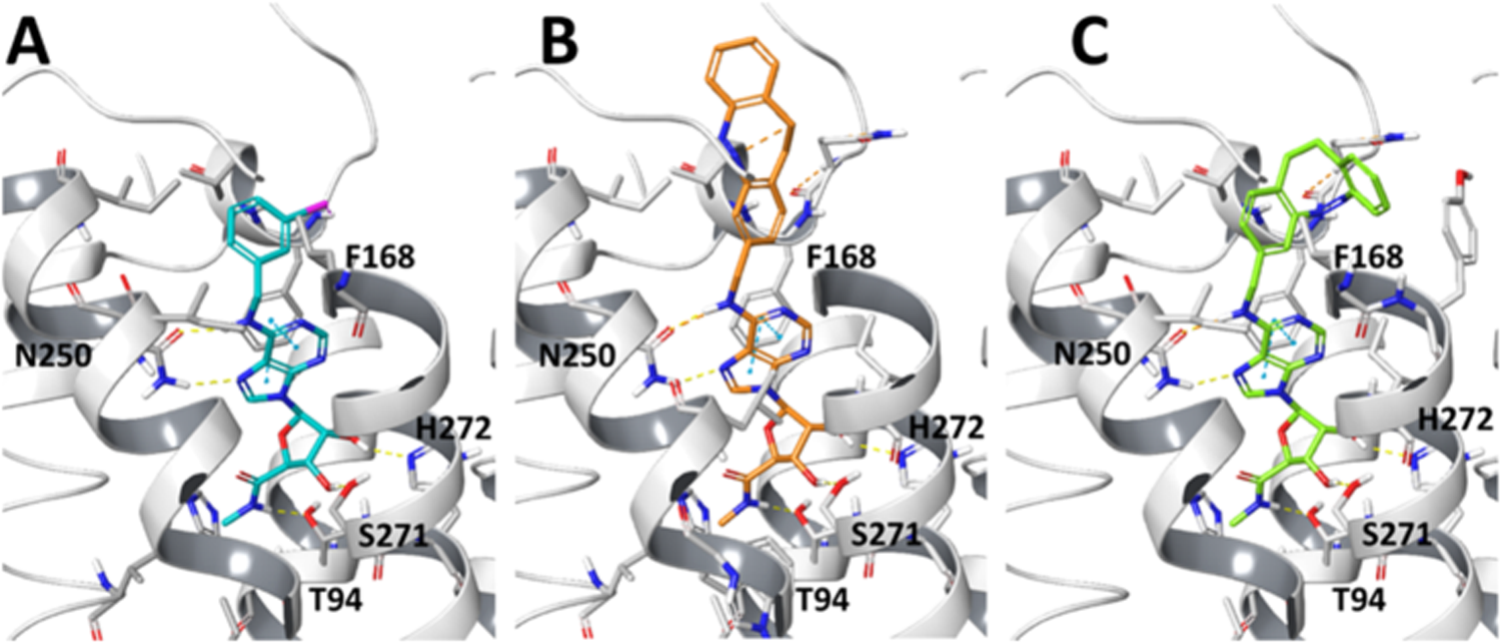
Docking of MRS7787 stereoisomers to a human A_3_R model. (A) Docking pose of IB-MECA (cyan) at the hA_3_R model obtained through induced fit docking. (B–C) Docking pose of an ***E***-MRS7787 (orange) and ***Z***-MRS7787 (green), respectively, at the optimized hA_3_R model. Hydrogen bonds are represented by yellow dashed lines, and clashes by orange dashed lines.

MRS7787 (Figure 1) is a nucleoside derivative based on the prototypical selective A_3_R agonist methyl 1-[*N*^6^-(3-iodobenzyl)-adenin-9-yl]-β-d-ribofuronamide (IB-MECA, also known as Piclidenoson), currently in clinical trials for psoriasis with demonstrated safety and efficacy at 16 weeks in an initial Phase III trial (Comfort)^17^ and was previously in a Phase III trial for rheumatoid arthritis.^18^ A hydrophobic *N*^6^-arylalkyl group is present in IB-MECA, which is common to various adenosine derivatives that act as receptor agonists, including those that show A_3_R selectivity. Interestingly, changing the nature and bulkiness of that substituent in an outward-facing extended conformation is documented to vary the maximal A_3_R efficacy, *i.e.*, magnitude of the functional response to an agonist (*E*_max_), independently of the agonist’s binding affinity.^19^ Therefore, we hypothesized that transformation of the favored *N*^6^-benzyl moiety of IB-MECA into a photoisomerizable diazocine group could alter its *E*_max_ and make it tunable upon visible light-induced *Z*–*E* interconversion (Figure 2A).

## Results

### Design of Photoswitchable Diazocine Derivatives for A_3_R

The design of diazocine-based A_3_R ligands began with the binding mode prediction of IB-MECA in an A_3_R model. Thus, an IB-MECA pose was obtained by induced fit docking in the theoretical A_3_R structure (Figure 2A), which was previously obtained by homology modeling.^20^ In this pose the agonist’s nucleosidic scaffold resembles adenosine bound to two other adenosine receptor subtypes, *i.e.*, A_2A_R and A_1_R, by making a π–π stacking with F168, a bidentate hydrogen bond with N250, and hydrogen bonds with T94, S271, and H272.

The 3-iodobenzyl moiety faces the receptor’s extracellular portion and provides a rationale for possible derivatization with dibenzodiazocine moieties. Two *E* and *Z* different stereoisomers were designed (Figure S1, Supporting Information) and docked at the refined receptor. An *E* and a *Z* stereoisomer could be docked at the A_3_R model maintaining a binding-mode similar to IB-MECA (Figure 2B,C), but with some clashes with the receptor and within the ligand. As a result, it was difficult to discriminate the binding capacity of the two compounds on the basis of the docking final states.

### Synthesis of MRS7787

**Figure 3:**
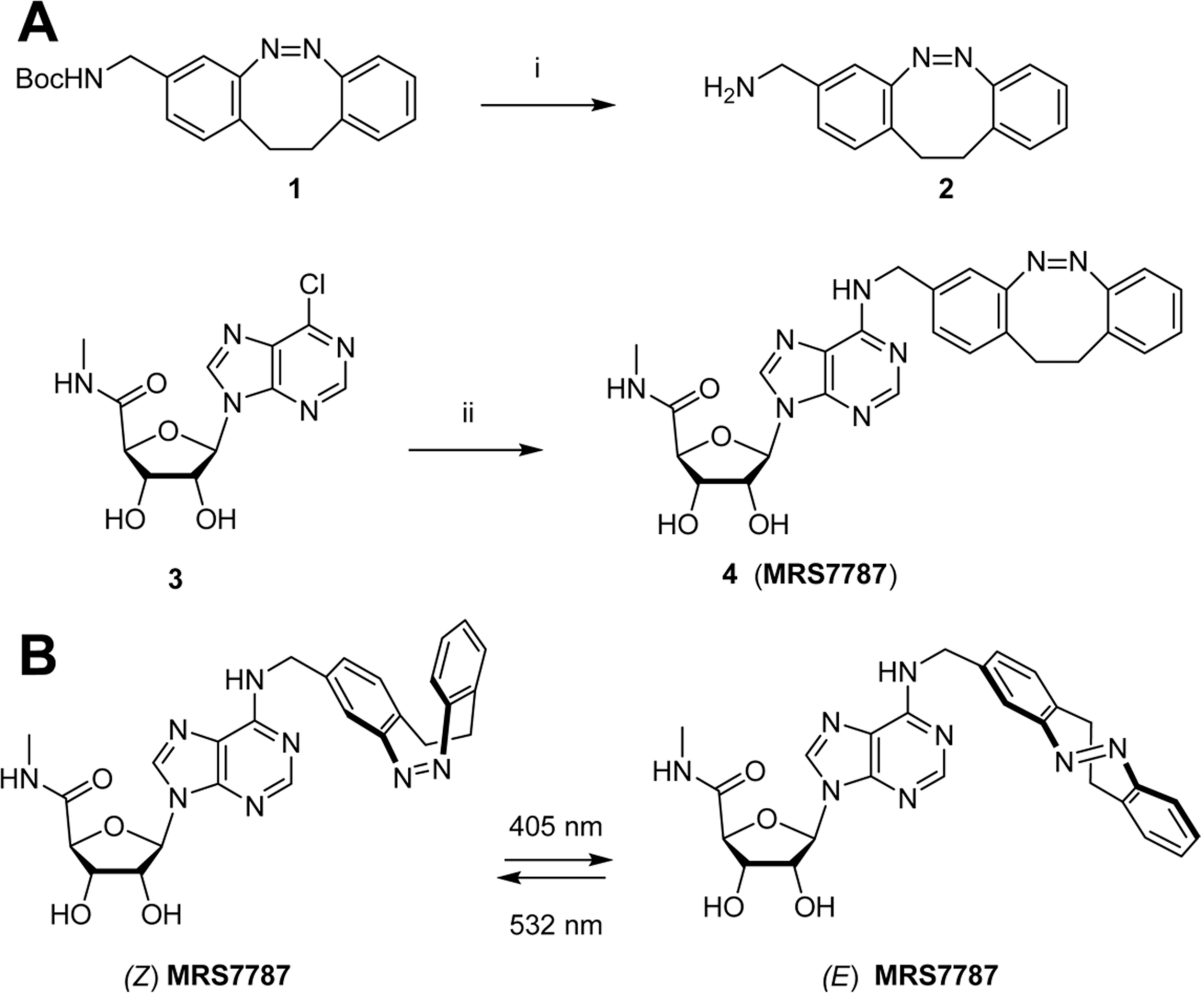
Chemical synthesis of MRS7787 **(4)**. (A) Conditions and reagents: (i) 4 N HCl in dioxane, CH_2_Cl_2_, rt, 1 h; (ii) **2**, DIPEA, 2-propanol, reflux, overnight. (B) Photoisomerization process between the *Z* and *E* states of MRS7787.

To synthesize MRS7787 (Figure 3), the diazocine Boc-amino-methyl precursor **1** was first generated based on previous reports.^21−23^ The 6-chloro precursor **3** of IB-MECA (Supporting Information) was then treated with the free amino (Boc-removed) diazocine intermediate **2** to yield the final product **4** (MRS7787).

### Photochemical Properties of MRS7787

**Figure 4:**
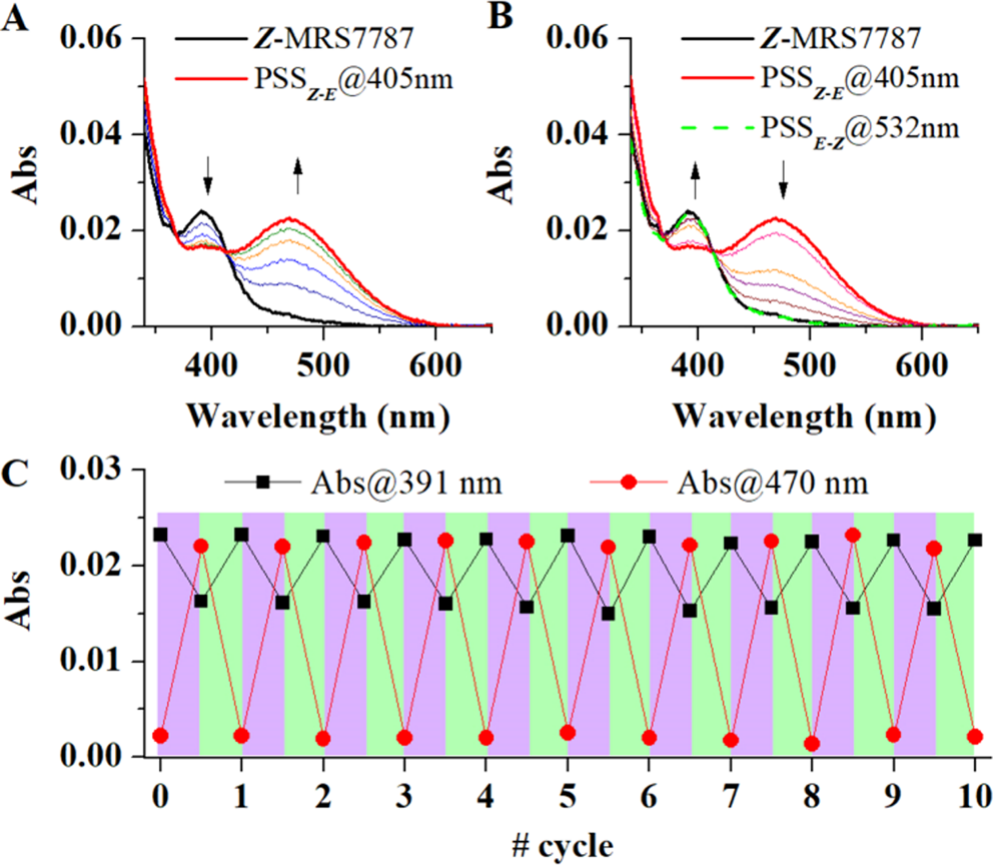
Photochemical characterization of MRS7787. (A) Variation of the absorption spectra of ***Z***-MRS7787 in PBS:DMSO 98:2 upon irradiation at 405 nm (30 s increments at 6.5 mW cm^–2^) and until a photostationary state (PSS_***Z**–**E***_) is obtained (*c*_MRS7787_ = 71 μM). (B) Variation of the absorption spectra of the PSS_***Z**–**E***_ of MRS7787 in PBS:DMSO 98:2 upon illumination at 532 nm (60 s at 10 mW cm^–2^) (*c*_MRS7787_ = 71 μM). For sake of comparison, the spectrum of the initial ***Z***-MRS7787 compound is shown, which matches the spectrum of the PSS_***E**–**Z***_ achieved after irradiation at 532 nm. (C) Absorbance variation at 391 (λ_max,***Z***-MRS7787_) and 470 nm (λ_max,***E***-MRS7787_) upon 10 consecutive cycles of ***Z***–***E*** photoisomerization of MRS7787 in PBS:DMSO 98:2 under sequential irradiation at 405 nm (30 s at 6.5 mW cm^–2^; violet) and 532 nm (60 s at 10 mW cm^–2^; green) (*c*_MRS7787_ = 71 μM).

We next characterized the photophysical properties of MRS7787 in aqueous buffer (PBS:DMSO 98:2) by means of UV–vis absorption measurements. As previously reported for other diazocine derivatives^2,5,21,24,25^ the as-synthesized ***Z***-MRS7787 isomer showed a defined absorption band at ca. 400 nm (λ_max_ = 391 nm), which can be ascribed to the *n* → π* transition of its bridged azobenzene chromophore. Irradiation of this band with violet light (λ_exc_ = 405 nm) led to spectral changes that are indicative of *Z* → *E* photoisomerization: a decrease in the absorption signal of ***Z***-MRS7787 and the appearance of a new red-shifted band at λ_max_ = 470 nm, typically associated with *E*-diazocines^2,5,21,24,25^ (Figure 4A).

Because of the separation between the *Z* and *E* isomers absorption peaks, this process was found to be rather efficient, as the photostationary state (PSS_*Z*–*E*_) generated under violet light irradiation contained 69 and 61% of ***E***-MRS7787 in CD_3_OD and PBS:DMSO 98:2, respectively (Figure S2, Supporting Information). Subsequent illumination with green light (λ_exc_ = 532 nm) resulted in quantitative *E* → *Z* photoisomerization and rapid recovery of the absorption signal of the initial ***Z***-MRS7787 compound (Figure 4B). By contrast, thermal *E* → *Z* back-isomerization in the dark occurred on a much longer time scale, and the half-life of ***E***-MRS7787 at physiological pH was measured to be *t*_1/2_ = 18.4 and 6.7 h at room (*T* = 22 °C) and physiological (*T*= 37 °C) temperatures, respectively (Figure S3, Supporting Information). Finally, the reversible photoswitching of MRS7787 was found to be robust and repetitive, as at least ten *Z*–*E* photoisomerization cycles could be registered upon sequential violet and green light irradiation without apparent degradation effects (Figure 4C).

### MRS7787 Is a Selective Photoswitchable A_3_R Agonist

**Figure 5:**
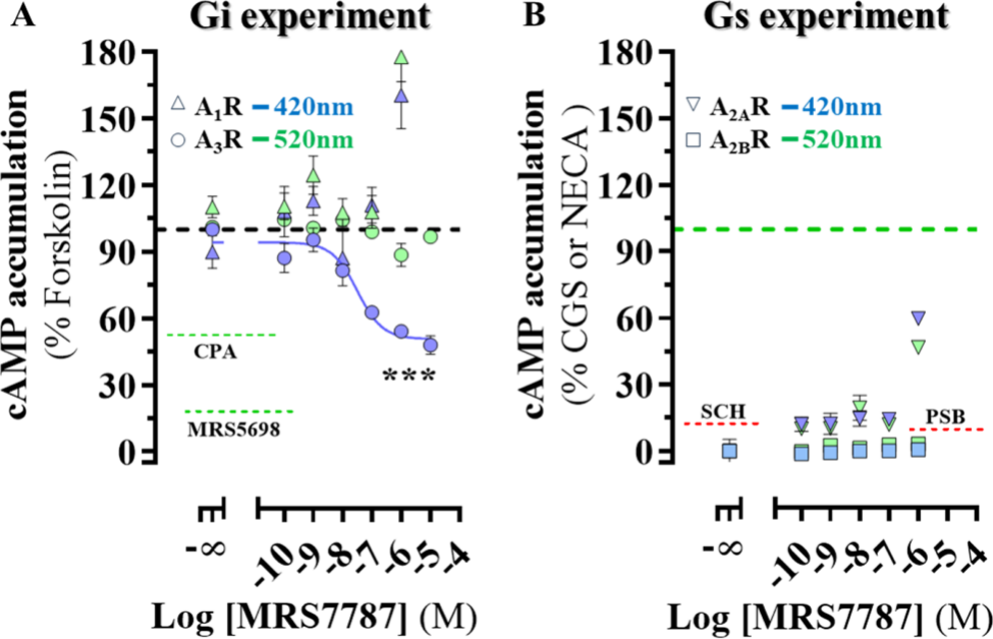
Photomodulation of the intrinsic activity of MRS7787 in living cells. HEK-293 cells expressing A_1_R, A_3_R, A_2A_R, and A_2B_R were challenged with increasing concentrations of MRS7787 irradiated with 420 nm (blue symbols) or 520 nm (green symbols) before cAMP accumulation was determined (see Supporting Information). In Gi stimulation experiments (A), forskolin (direct activator of adenylate cyclase) treatment induced maximum cAMP accumulation (black dashed line) and treatment with MRS5698 and CPA (*N*^6^-cyclopentyladenosine, selective A_1_R agonist) induced the maximum A_3_R- and A_1_R-mediated inhibition of cAMP accumulation (respectively green dashed line). In Gs stimulation experiments (B) treatment with CGS21680 and NECA induced maximum stimulation of cAMP accumulation in cells expressing A_2A_R and A_2B_R, respectively (green dashed line). Treatment with SCH442416 (selective A_2A_R antagonist) and PSB603 (selective A_2B_R antagonist) determined the blockade of A_2A_R, and A_2B_R, respectively. Data are expressed as the mean ± SEM of three independent experiments performed in quadruplicate. ***P < 0.0001, *F*_(6,238)_ = 80 when all nonlinear regression EC_50_ and *E*_max_ values from were compared.

Photomodulation of the intrinsic activity of MRS7787 was evaluated by monitoring A_3_R-mediated inhibition of cyclic adenosine monophosphate (cAMP) accumulation in a cell line expressing the receptor. As expected, activation of A_3_R by a prototypical highly selective agonist (*i.e.*, (1*S*,2*R*,3*S*,4*R*,5*S*)-4-(6-((3-chlorobenzyl)amino)-2-((3,4-difluorophenyl)-ethynyl)-9*H*-purin-9-yl)-2,3-dihydroxy-*N*-methylbicyclo[3.1.0]hexane-1-carboxamide, MRS5698) induced a robust cAMP accumulation response (Figure 5A). Interestingly, although the photosensitive MRS7787 compound was unable to inhibit cAMP accumulation in its initial ***Z***-configuration (or 520 nm-induced state), it elicited, conversely, a partial agonist concentration–response (IC_50_ = 31 nM; CI_95_ = 1.9–52 nM; E_max_ = 62 %; CI_95_ = 54–68 %) when the ***E***-configuration (or 420 nm-induced state) was generated (Figure 5A). The selectivity of MRS7787 was evaluated in cells stably expressing A_1_R, A_2A_R, and A_2B_R. Both ***Z***-MRS7787 and ***E***-MRS7787 failed to elicit A_1_R-mediated inhibition of forskolin-stimulated cAMP accumulation. However, a light independent and low-potency effect (>1 μM) on forskolin-stimulated cAMP accumulation—indicative of A_1_R-Gs protein coupling—was observed (Figure 5A), as previously described in other heterologous overexpressing systems.^26^ Furthermore, ***Z***-MRS7787 and ***E***-MRS7787 were unable to promote activation of the two G_s_ protein-coupled adenosine receptors, A_2A_R and A_2B_R, at reasonable (<1 μM) concentrations (Figure 5). Therefore, this shows that the inhibitory response to cAMP accumulation promoted by ***E***-MRS7787 could be mainly attributed to an A_3_R activation-dependent intracellular cascade signaling pathway.

### MRS7787 Photoactivates A_3_R in a Mouse Model of Psoriasis

**Figure 6:**
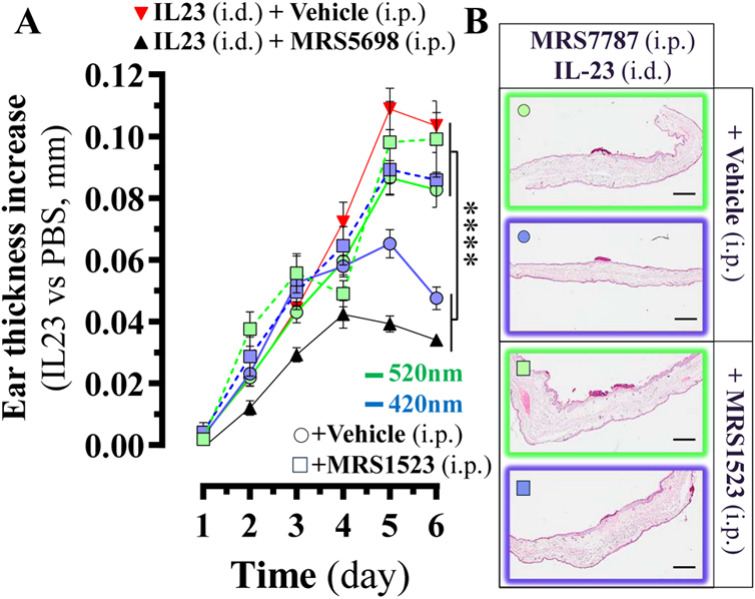
MRS7787 photoactivates A_3_R in a mouse model of psoriasis. (A) The temporal drug treatment of the IL-23-induced psoriasis model is explained in Figure S5 (Supporting Information). IL-23 treated (i.d., intradermal) mice were administered (i.p., intraperitoneal) with vehicle (red triangles) or MRS5698 (1 mg/kg; black triangles). IL-23 administered animals were treated with MRS7787 (1 mg/kg) in the absence (circles) or presence (squares) of MRS1523 before both ears were irradiated with 420 nm (blue line) or 520 nm (green line) for 8 min. Ear thickness was measured in millimeters (mm) and shown as mean ± S.E.M., *n* = 7–13 mice per group. ****P < 0.0001 one-way ANOVA with Dunnett’s posthoc test comparing to MRS5698. (B) Representative H&E-stained ear sections of IL-23-treated mice from the indicated experimental group. Scale bar = 500 μm.

Next, we sought to determine whether MRS7787 can control IL-23-associated inflammatory responses associated with proinflammatory cytokine interleukin (IL)-23 in the mouse model of psoriasis. IL-23 injection into the mouse ear induced marked swelling compared to contralateral PBS-administered mice (Figure S4, Supporting Information), as previously reported.^16^ Additionally, light irradiation, irrespective of the wavelength used, did not influence IL-23-induced inflammation (Figure S4, Supporting Information). Noticeably, systemic administration of MRS5698 prevented IL-23-induced ear inflammation (Figure 6), as reported for A_3_R agonism.^16^ Conversely, under the same time-course and experimental conditions (Figure S5, Supporting Information), MRS7787 was unable to preclude the IL-23-induced psoriatic-like phenotype in its initial ***Z***-state (or 520 nm light-irradiated; Figure 6A). Thus, the inactivity suggested that the molecule had a configuration-dependent reduced molecular interaction within the A_3_R. Importantly, when the ears of mice administered with MRS7787 were irradiated with 420 nm light at days 3, 4, and 5, a significant reduction in the IL-23-induced ear thickness was observed at day six of the experiment (Figures 6A and S6A). Also, epidermis (Figure 6B, light purple stain) was reduced at day 6 (Figure S6B), as well as a moderate effect on hyperkeratosis (Figure 6B, pink layer on the outermost part of the epidermis; and Figure S6C) was observed, in agreement with a reduced inflammatory response. Importantly, treatment of the animals with the selective A_3_R antagonist, MRS1523, precluded all these anti-inflammatory effects of ***E***-MRS7787 (Figures 6and S6). Thus, the photoconversion of MRS7787 to the ***E***-state appeared to be robust in a physiological environment. Taken together, these results demonstrate the photoconversion of MRS7787 intradermally upon noninvasive topical irradiation of the mouse ear, thus promoting the selective A_3_R targeting locally and attenuating IL-23-induced inflammation.

## Conclusions

Photopharmacology holds promise for improving therapeutic outcomes while reducing side effects. However, its translation into clinical applications is currently limited. To progress toward this direction, two important issues must be considered: (i) the design of photopharmaceuticals with precise mechanisms of action and optimized photoactivation properties that can safely and effectively interact with biological systems; and (ii) the development of light sources compatible with existing medical practices. Despite these challenges, significant progress has been made in recent years.^1^ Our dark-adapted (pharmacologically inactive) photoswitchable compound exemplifies the potential of combining diazocine-based photodrugs with light-dependent antipsoriatic efficacy upon noninvasive topical skin irradiation, thus increasing its therapeutic possibilities. Interestingly, phototherapy, either in the absence or presence of systemic treatments, has been successfully used for decades to treat patients with either mild, moderate, or severe plaque psoriasis.^27^ Thus, we reasoned that exploring potential multimodal treatment paradigms combining conventional phototherapy with photopharmacology might provide synergistic effects, enhance therapeutic efficacy, and provide a more comprehensive solution for patients facing treatment challenges (*i.e.*, resistant psoriasis).

## Supporting Information Available

Details of drug synthesis; general materials and methods; biological data; analytical data (PDF)
