# Supplementary material for: Photoswitchable Diazocine Derivative for Adenosine A3 Receptor Activation in Psoriasis
Source: J Am Chem Soc. 2024 Dec 16;147(1):874–9. doi: 10.1021/jacs.4c13558 (PMC11726555; doi:10.1021/jacs.4c13558)
Supplement: Supplementary file 1 — ja4c13558_si_001.pdf [file ja4c13558_si_001.pdf]

Supporting Information

Photoswitchable diazocine derivative for adenosine A_3 receptor activation in psoriasis

Marc López-Cano[1,2‡], Mirko Scortichini[3‡], Dilip K Tosh[3‡], Veronica Salmaso[3], Tongil Ko[4], Glòria Salort[1,2], Ingrid Filgaira[5,6], Concepció Soler[5,6], Dirk Trauner[4,7], Jordi Hernando[8*], Kenneth A. Jacobson[3*] and Francisco Ciruela[1,2*]

[1]Pharmacology Unit, Department of Pathology and Experimental Therapeutics, Faculty of Medicine and Health Sciences, Institute of Neurosciences, University of Barcelona, L'Hospitalet de Llobregat 08907, Spain. [2]Neuropharmacology and Pain Group, Neuroscience Program, Bellvitge Biomedical Research Institute, L'Hospitalet de Llobregat 08907, Spain. [3]Molecular Recognition Section, Laboratory of Bioorganic Chemistry, NIDDK, National Institutes of Health, Bethesda, Maryland 20892, United States. [4]Department of Chemistry University of Pennsylvania College of Arts and Sciences, Philadelphia, Pennsylvania 19104, United States. [5]Immunology Unit, Department of Pathology and Experimental Therapeutics, Faculty of Medicine and Health Sciences, University of Barcelona, L'Hospitalet de Llobregat 08907, Spain. [6]Immunity, Inflammation and Cancer Group, Oncology Program, Bellvitge Biomedical Research Institute, L'Hospitalet de Llobregat 08907, Spain. [7]Department of Chemistry, New York University, New York City, New York 10003, United States.[8]Department of Chemistry, Autonomous University of Barcelona, Cerdanyola del Vallès 08193, Spain.

‡Contributed equally to this work
*Corresponding authors

Table of Contents

	Page
**Synthesis of MRS7787**	
Materials and instrumentation	S3
General information and synthetic procedures	S4-S5
**Other Methods**	
Molecular modeling	S6-S7
Photochemical characterization	S7
Cell culture and stable transfection	S7-S8
cAMP accumulation inhibition assay	S8-S9
Animals	S9-S10
IL23-induced psoriatic-like phenotype	S10
Histochemistry	S10-S11
Statistical analysis	S11
**Analytical Data**	
NMR Spectra	S12-S32
Table S1. Summary of NMR spectra	S33-S34
MS, HPLC and IR data of MRS7787	S35-S38
Figure S1. Different stereoisomers of the diazocine moiety	S39
Figure S2. Photostationary mixtures of MRS7787 under irradiation	S40
Figure S3. Effect of temperature on the half-life of *E*-MRS7787	S41
**Biological Data**	
Figure S4. Effect of light on IL-23-induced psoriatic phenotype	S42
Figure S5. Temporal scheme for IL-23-induced mouse model of psoriasis	S43
Figure S6. Quantification of *E*-MRS7787-mediated anti-inflammatory effects in the mouse psoriasis-like model	S44
**References**	S45

Synthesis of MRS7787

Materials and instrumentation. All reagents and solvents were purchased from Sigma-Aldrich (St. Louis, MO). ^{1}H NMR spectra were obtained with a Bruker 500 spectrometer using $CDCl_3$, CD_3OD and DMSO as solvents. Chemical shifts are expressed in δ values (ppm) with tetramethylsilane (δ 0.00) for $CDCl_3$ and water (δ 3.30) for CD_3OD. NMR spectra were collected with a Bruker AV spectrometer equipped with a z-gradient [^{1}H, ^{13}C, ^{15}N]-cryoprobe. TLC analysis was carried out on glass sheets precoated with silica gel F254 (0.2 mm) from Aldrich. The purity of final nucleoside derivatives was checked using a Hewlett−Packard 1100 HPLC equipped with a Zorbax SB-Aq 5 μm analytical column (50 × 4.6 mm; Agilent Technologies Inc., Palo Alto, CA). Mobile phase: linear gradient solvent system, 5 mM TBAP (tetrabutylammonium dihydrogenphosphate):CH_3CN from 80:20 to 0:100 in 13 min; the flow rate was 0.5 mL/min. Peaks were detected by UV absorption with a diode array detector at 230, 254, and 280 nm. All derivatives tested for biological activity showed >95% purity by HPLC analysis (detection at 254 nm). Low-resolution mass spectrometry was performed with a JEOL SX102 spectrometer with 6-kV Xe atoms following desorption from a glycerol matrix or on an Agilent LC/MS 1100 MSD, with a Waters (Milford, MA) Atlantis C18 column. High resolution mass spectroscopic (HRMS) measurements were performed on a proteomics optimized Q-TOF-2 (Micromass-Waters) using external calibration with polyalanine, unless noted. Observed mass accuracies are those expected based on known performance of the instrument as well as trends in masses of standard compounds observed at intervals during the series of measurements. Reported masses are observed masses uncorrected for this time-dependent drift in mass accuracy. IR spectra was recorded with Bruker Alpha II compact FT-IR spectrometer.

General information. MRS7787 (**4**) was synthesized by the route shown in Figure 2. All subsequent reactions with diazocene derivatives were performed in a darkened room, and the reaction vessel was protected from light to avoid compound degradation. The Boc-aminomethyl derivative of diazocene **1** was synthesized as reported.[1–3] Boc deprotection of compound **1** with 4N HCl in dioxane and dichloromethane at room temperature provided the diazocine derivative **2**, which was used directly for next step without purification. Compound **3** was prepared following the reported procedure.[4] Reaction of the adenosine precursor **3** with the crude amino-methyl-diazocene intermediate **2** in isopropanol in presence of diisopropylethyl amine under reflux condition afforded the novel nucleoside derivative MRS7787 (**4**).

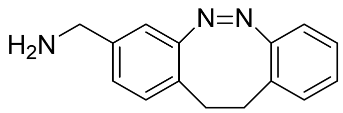

2

(Z)-(11,12-Dihydrodibenzo[c,g][1,2]diazocin-3-yl)methanamine (2)

4N HCl in dioxane (5 mL) was added dropwise to a solution of *tert*-butyl (*Z*)-((11,12-dihydrodibenzo[*c*,*g*][1,2]diazocin-3-yl)methyl)carbamate **1** (250 mg, 0.74 mmol) in dichloromethane (15 mL). and stirred for one hour at room temperature. The reaction was monitored by mass spectrometry. After completion of starting material, solvent was evaporated under vacuum and the resulting crude product **2** was directly subjected to next step without further purification. HRMS calcd $C_{15}H_{16}N_3$ $[M+H]^+$: 238.1344; found 238.1347.

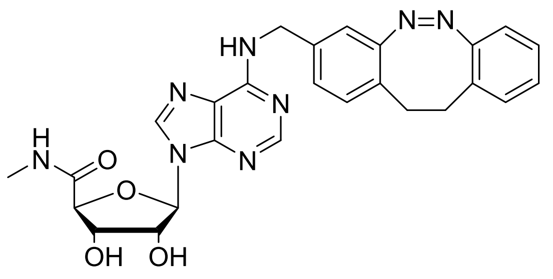

MRS7787 (**4**)

(2S,3S,4R,5R)-5-(6-((((Z)-11,12-dihydrodibenzo[c,g][1,2]diazocin-3-yl)methyl)amino)-9H-purin-9-yl)-3,4-dihydroxy-N-methyltetrahydrofuran-2-carboxamide (4)

The crude compound (Z)-(11,12-dihydrodibenzo[c,g][1,2]diazocin-3-yl)methanamine **2** (170 mg) and DIPEA (0.27 mL, 1.59 mmol) was added to a solution of compound **3** (100 mg, 0.32 mmol) in 2-propanol and reflux for 3 hours. Solvent was evaporated under vacuum and the residue was purified on flash silica gel column chromatography (CH_2Cl_2:MeOH = 25:1) to afford the N^6-diazocine substituted nucleoside derivative MRS7787 [**4**] (88 mg, 54%) as a glassy solid. IR ν_{max}: 3253 cm^{-1}, 1619 cm^{-1}; ^{1}H NMR (500 MHz, MeOD): δ 8.28 (1H, s), 8.24 (1H, s), 7.10 (1H, m), 7.06 (1H, dd, $^3J_{HH}$ = 7.9 *Hz*, $^4J_{HH}$ = 1.7 *Hz*), 7.00 (1H, d, $^3J_{HH}$ = 8.3 *Hz*), 7.01 (2H, m), 6.82 (1H, d, $^4J_{HH}$ = 1.3 *Hz*), 6.74 (1H, d, $^3J_{HH}$ = 7.8 *Hz*), 6.01 (1H, d, $^3J_{HH}$ = 7.7 *Hz*), 4.74 (1H, dd, $^3J_{HH}$ = 7.7 *Hz*, $^3J_{HH}$ = 4.8 *Hz*), 4.72 (2H, br. s), 4.48 (1H, d, $^3J_{HH}$ = 1.1 *Hz*), 4.32 (1H, dd, $^3J_{HH}$ = 4.8 *Hz*, $^3J_{HH}$ = 1.4 *Hz*), 2.87 (3H, s, overlapping), 2.78-2.92 (4H, multiplet, overlapping). ^{13}C NMR (125 MHz, MeOD): δ 172.96, 156.80, 156.87, 156.36 (br.), 149.64 (br.), 142.40, 139.56 (br.), 131.30, 130.99, 129.77, 128.69, 128.47, 127.96, 127.52, 121.77 (br.), 119.76, 118.68, 90.68, 86.65, 75.10, 73.52, 44.41 (br.), 32.38, 32.54, 26.15. ^{15}N NMR (50 MHz, MeOD): δ 541, 540, 231, 226, 219, 165, 107. HRMS calcd $C_{26}H_{27}N_8O_4$ $[M+H]^+$: 515.2158; found 515.2155.

Other Methods

Molecular modeling

The structure of human A_3R was reported in a previous work,[5] where it was obtained by homology modeling using as templates an agonist-bound intermediate state $A_{2A}R$ X-ray structure (3QAK[6] and 4UHR[7]) and an antagonist-bound A_1R (5UEN[8]) X-ray structure for the tip of TM2. The structure was subjected to the Protein Preparation Wizard[9] tool of the Schrödinger[10] suite, and the following tautomeric states were assigned to histidine: HSD 272 and HSE 79, 95, 124, 158 (according to CHARMM nomenclature).

IB-MECA was docked using the induced fit docking[11,12] procedure of the Schrödinger[10] suite, with the following settings. N250 and F168 were used to define the center of the grid, with an inner box of 10 Å and an outer box of 30 Å. Residues within 5 Å from the ligand were refined, and the Glide-XP scoring function was adopted for the redocking phase. Enhancement planarity of conjugated pi groups was employed during conformational sampling. The second ranked pose was selected, because it maintained key hydrogen bonds with N250, T94, S271 and H272. The protein structure refined by induced fit docking was extracted for further docking of MRS7787.

The *E* and *Z* MRS7787 stereoisomers were drawn starting from the coordinates of dibenzodiazocine molecules extracted from the Cambridge Structural Database[13] (IDS: 1558112 (*E*), 1992126 (*E*), 757546 (*Z*)). The MRS7787 stereoisomers were docked at the A_3R putative orthosteric binding site using Glide[14,15]-XP[16], using F168 and N250 to define the center of the grid, with inner and outer dimensions of 10 Å and 30 Å, respectively. The following settings were added to the default ones: sampling of nitrogen inversion and of ring conformations was adopted (including the initial ring conformation), and enhanced planarity of conjugated pi groups was employed. The post-docking minimization stage was included, and the strain correction term was applied. A maximum of 20 poses per compound was

generated. The first poses maintaining the typical pattern of interactions with ARs were selected.

Photochemical characterization

All photochemical experiments were performed using HPLC-quality solvents. Steady-state UV-vis absorption measurements were recorded in PBS:DMSO 98:2 on a HP 8453 spectrophotometer with temperature control, which allowed us monitoring the photoinduced interconversion between the two isomers of MRS7787 as well as its thermal *E* → *Z* back-isomerization in the dark. The composition of the photostationary states (PSS) obtained upon irradiation of MRS7787 PBS:DMSO 98:2 was determined from its UV-vis absorption spectrum and those of the as-synthesized *Z*-MRS7787 and the photoinduced *E*-MRS7787 pure compounds. The latter was estimated from the absorption spectrum of a well-known mixture of the *Z* and *E* isomers of MRS7787, the composition of which was measured by ^{1}H NMR in CD_3OD. Different excitation sources were used in the photochemical experiments: a LED405 ($\lambda_{exc,max}$ = 405 nm, Chanzon) for *Z* → *E* photoisomerization (6.5 mW cm^{-2}), and the second harmonic of a ns-pulsed Nd:YAG laser (λ_{exc} = 532 nm, Brilliant, Quantel) for *E* → *Z* photoisomerization (10.0 mW cm^{-2}).

Cell culture and stable transfection.

Human embryonic kidney 293 (HEK-293) cells obtained from ATCC (American Type Culture Collection, Rockville, MD, USA; CRL-321, RRID: CVCL_0063) were grown in Dulbecco's modified Eagle's medium (DMEM) pre-heated at 37°C and supplemented with: 5% (v/v) fetal bovine serum (previously inactivated at 55°C for 30 min), 100 U/mL penicillin, 100 µg/mL streptomycin, 2 mM L-glutamine and non-essential amino acids. Manipulation and maintenance were carried out in a biological safety cabinet class 1 and in an incubator at 37°C,

5% CO_2 and 90% relative humidity. The absence of mycoplasma was checked regularly, thus only mycoplasma-free cells were used. HEK-293 cells were transfected with 10 μg of the corresponding pIRES-SP-HA-NL-AR plasmid (obtained from GenScript, Leiden, Netherlands) encoding for each human adenosine receptors (hAR): A_1R, $A_{2A}R$, $A_{2B}R$ or A_3R plus the signal peptide of metabotropic glutamate receptor type 5 (i.e., SP), influenza hemagglutinin peptide (i.e., HA) and NanoLuc protein (i.e., NL). Transfection was performed by using polyethylenimine (PEI) transfection reagent.[17] After transfection, cells growing in 60 cm^2 plates were supplemented DMEM in the presence of 2 mg/ml geneticin for 3 weeks to select cells expressing the corresponding hAR, thus ensuring its stable expression, as previously described.[18,19]

cAMP accumulation inhibition assay

cAMP accumulation was measured using the LANCE Ultra cAMP kit following the manufacturer's indications, as previously described.[19] Briefly, cells (100-400 cells/μL) were first incubated with 2 ml of 0.1% BSA, adenosine deaminase (ADA, 0.5 U/ml) and zardaverine (2 μM) in serum-free supplemented DMEM, namely stimulation buffer, for 1 hour at 37 ° C and double orbital agitation at 300 rpm. Subsequently, stimulation buffer was removed, and cells were incubated with fresh stimulation buffer (vehicle, DMEM:DMSO 999:1) or increasing concentrations of MRS7787 (in vehicle solution), which was previously light irradiated with 420 or 520 nm wavelength light at 1.18 mW/cm^2 and 7.64 mW/cm^2 intensity LED, respectively. In the case of Gi stimulation experiments (i.e., A_1R and A_3R), cells were challenged with forskolin (1 μM) plus MRS7787 for 30 min. Otherwise, in the case of Gs stimulation experiments (i.e., $A_{2A}R$ and $A_{2B}R$), cells were only incubated with MRS7787. Subsequently, treated cells were placed in a 384-well plate and Eu-cAMP tracer and ULightTM anti-cAMP reagents were added. Subsequently, cells were incubated for 1 h in the dark at 22^0C.

Finally, measurements at 620 and 665 nm were performed in a CLARIOstar Plus multimode microplate reader to determine cAMP levels following the manufacturer's instructions. The results were expressed as the percentage of activation of each hAR induced by the drug treatment following the equation:

$$\text{cAMP accumulation (\%)} = [FV_{drug} / FV_{max}] \times 100$$

Where FV_{drug} represent the FRET value in the presence of drug + forskolin (i.e., Gi experiments) or drug alone (i.e., Gs experiments). On the other hand, FV_{max} represents the maximum FRET value obtained in each experimental condition: i) Gi stimulation experiments, forskolin 1µM; ii) Gs stimulation experiments, CGS21680 (50 nM) for $A_{2A}R$ and NECA (1µM) for $A_{2B}R$ experiments. Furthermore, in Gi stimulation experiments selective A_3R and A_1R agonists (i.e., 1µM MRS5698 and 100 nM CPA, respectively) were used to determine agonist-mediated cAMP accumulation. Similarly, in Gs stimulation experiments selective $A_{2A}R$ and $A_{2B}R$ antagonists (i.e., 1µM SCH442416 and 1µM PSB603, respectively) were used to determine the blockade of agonist-mediated cAMP accumulation. Concentration-response curves were fitted by non-linear regression using GraphPad Prism 10.3.1 (GraphPad Software, La Jolla, CA, USA).

Animals

Adult C57BL/6N (Envigo Rms Spain SL., Sant Feliu de Codines, Spain) female mice bred in the animal facility of University of Barcelona (Campus of Bellvitge) aging 12-14 weeks-old and weighing 25-35 g were used. The University of Barcelona Committee on Animal Use and Care (CEEA) approved the protocol and experiments were conducted under the authorization of the Catalan Government (196/22). Following the approved experimental protocol all animals were supervised daily to assess signs of adverse effects during treatment. A retrospective analysis of the protocol demonstrated that no corrective measures (i.e., use of analgesics) were

needed. Animals were housed and tested in compliance with the guidelines provided by the Guide for the Care and Use of Laboratory Animals[20] and following the European Union directives (2010/63/EU). Mice were housed in groups of five in standard cages with *ad libitum* access to food and water and maintained under a 12 h dark/light cycle (starting at 7:30 AM), at 22 °C and 66% humidity (standard conditions).

IL23-induced psoriatic-like phenotype

The IL23-induced mouse model of psoriasis was performed using C57BL/6N mice, as previously described. The experimental approach consisted in a 6 consecutive days protocol. Briefly, mice were anesthetized by the i.p. administration of a ketamine (100 mg/kg)/xylazine (10 mg/kg) mixture, and ear thickness was measured using a digital calliper. Then recombinant mouse IL23 (500 ng) or PBS were i.d. injected into the ears by using a Hamilton syringe during four consecutive days (days 1, 2, 3, 4 and 5). At days 3, 4 and 5 animals were treated intraperitoneally (i.p.) with vehicle (14.2% DMSO and 14.2% Tween80 in saline) or drugs (i.e., MRS5698 or MRS7344; 1mg/Kg) 20 min before being anaesthetized. Light irradiation of the corresponding ear was conducted before IL-23 administration by using a custom-made 9 × 4 light-emitting diode (LED) matrix (12 × 9 cm) placed at a height of 8 cm above the head of the animals. Following that approach, a 420 nm or 520 nm wavelength light regime consisting of continuous light-irradiation at 1.18 mW/cm^2 or 7.64 mW/cm^2 intensity LED respectively, was performed during 8 min. The contralateral ear was covered to be protected from collateral light-irradiation.

Histochemistry

Tissue samples from mice were fixed with 4% paraformaldehyde in PBS, embedded in paraffin, and then cut in 5 μm sections. Sample processing for hematoxylin and eosin (H&E)

staining was performed according to standard procedures.[21] Images were captured using a Zeiss AXIO Observer 7 inverted fluorescence motorized XY microscope and analyzed with the FIJI open-source platform for biological image analysis. Total ear thickness and epidermal thickness were determined by measuring the length of straight lines drawn perpendicular from the basal layer to the stratum corneum in five randomly selected fields per section, as previously described.[22] Hyperkeratosis was quantified as the percentage of the affected (lesioned) skin length relative to the total skin length showed in each image.

Statistical analysis

GraphPad Prism 10.3.1 (San Diego, CA, USA) software was used for statistical analysis. Statistical significance was accepted when $P < 0.05$. Data are represented as mean ± standard error of mean (SEM). The number of samples/animals (n) in each experimental condition is indicated in the corresponding figure legend. Outliers were assessed by the ROUT method[23] assuming a Q value of 1% in GraphPad Prism 10.3.1. No animals were excluded. Comparisons among experimental groups were performed by one-way analysis of variance (ANOVA) followed by Dunnett's multiple comparison post-hoc test.

Analytical data:

NMR spectra of compound MRS7787 (4)

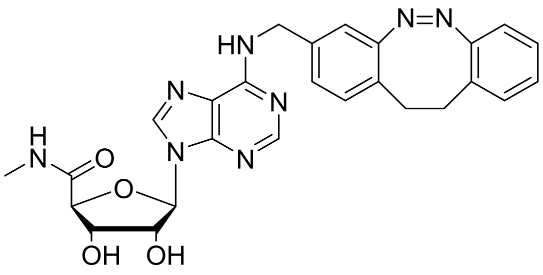

MRS7787 (**4**)

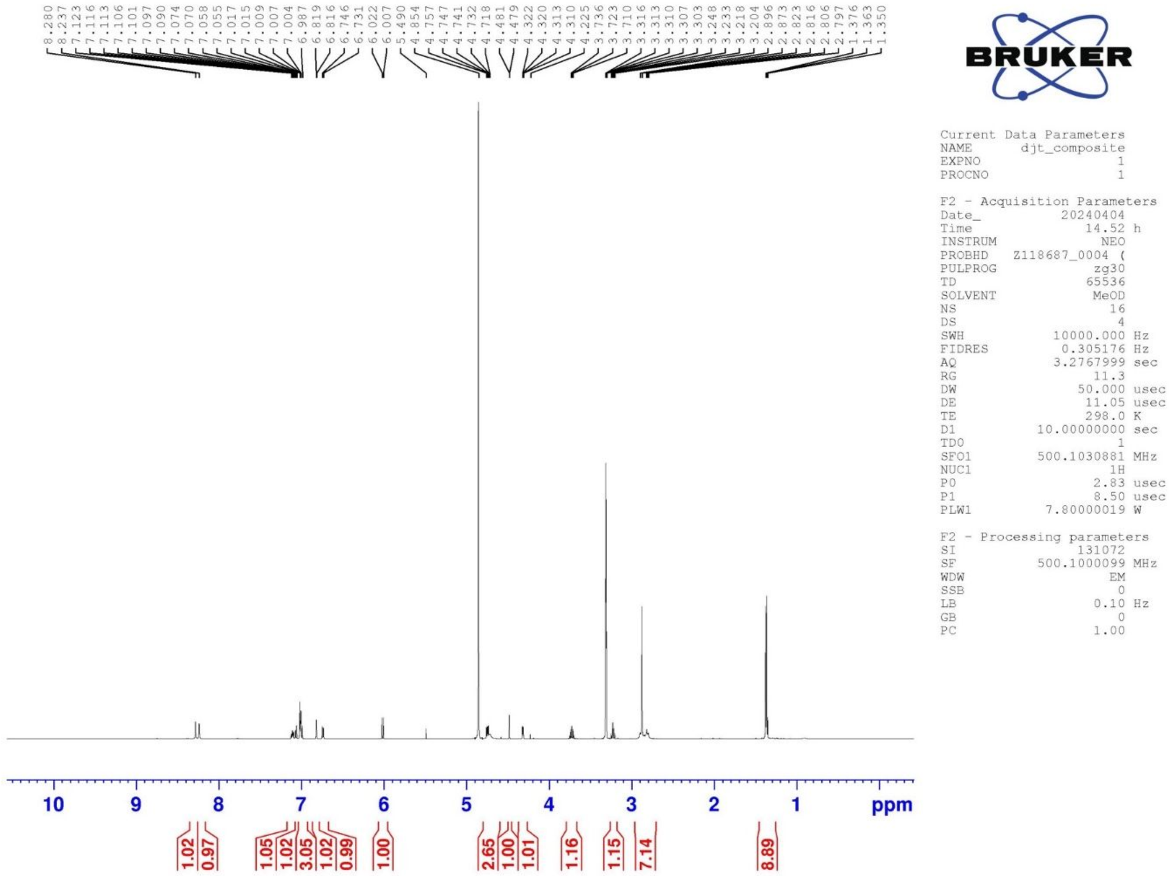

^{1}H NMR (500 MHz, MeOD)

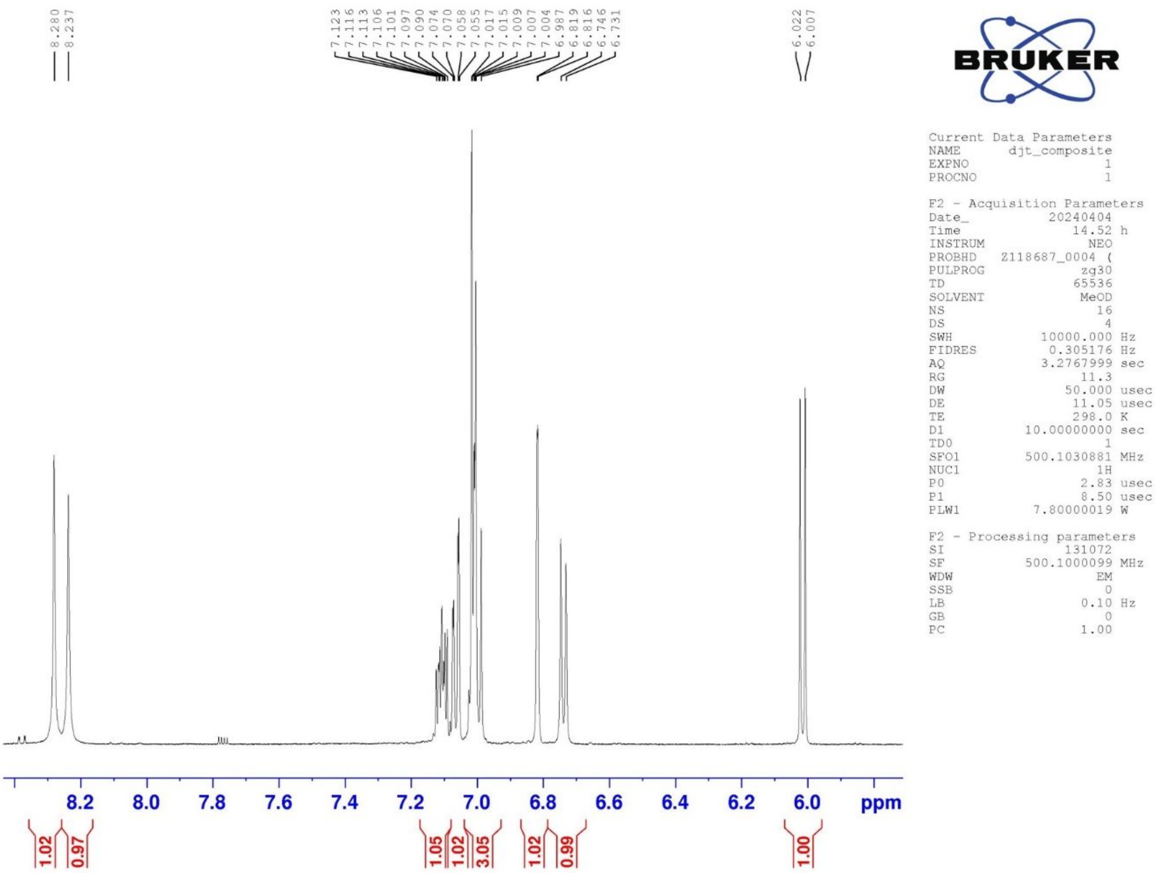

^{1}H NMR (500 MHz, MeOD) (expanded region)

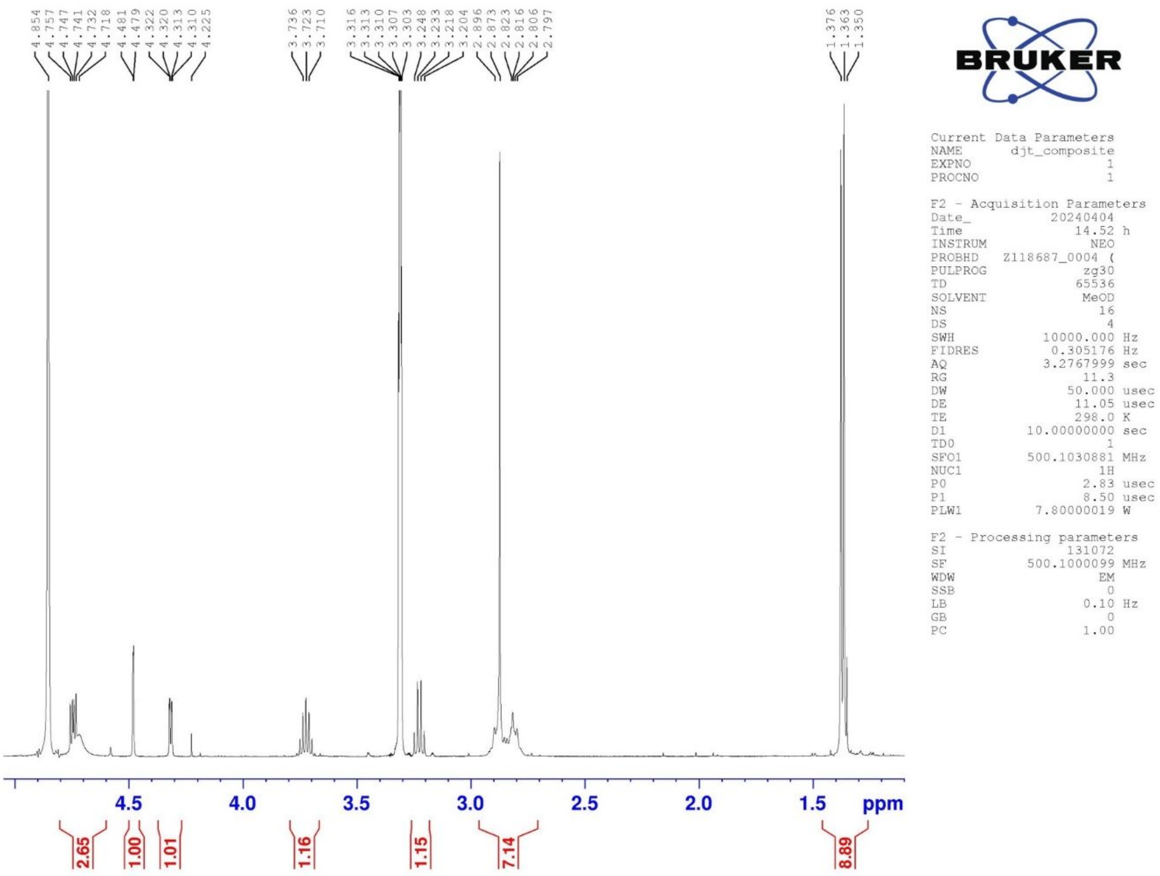

^{1}H NMR (500 MHz, MeOD) (expanded region)

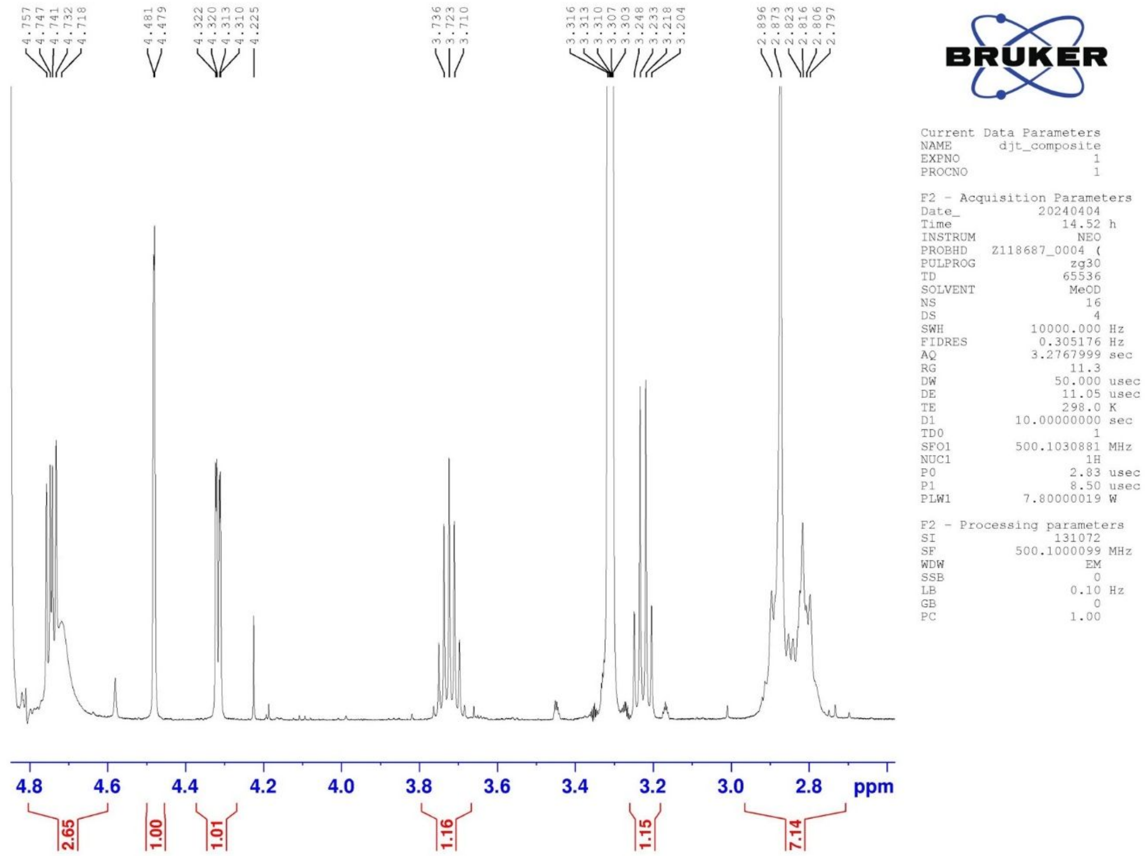

^{1}H NMR (500 MHz, MeOD) (expanded region)

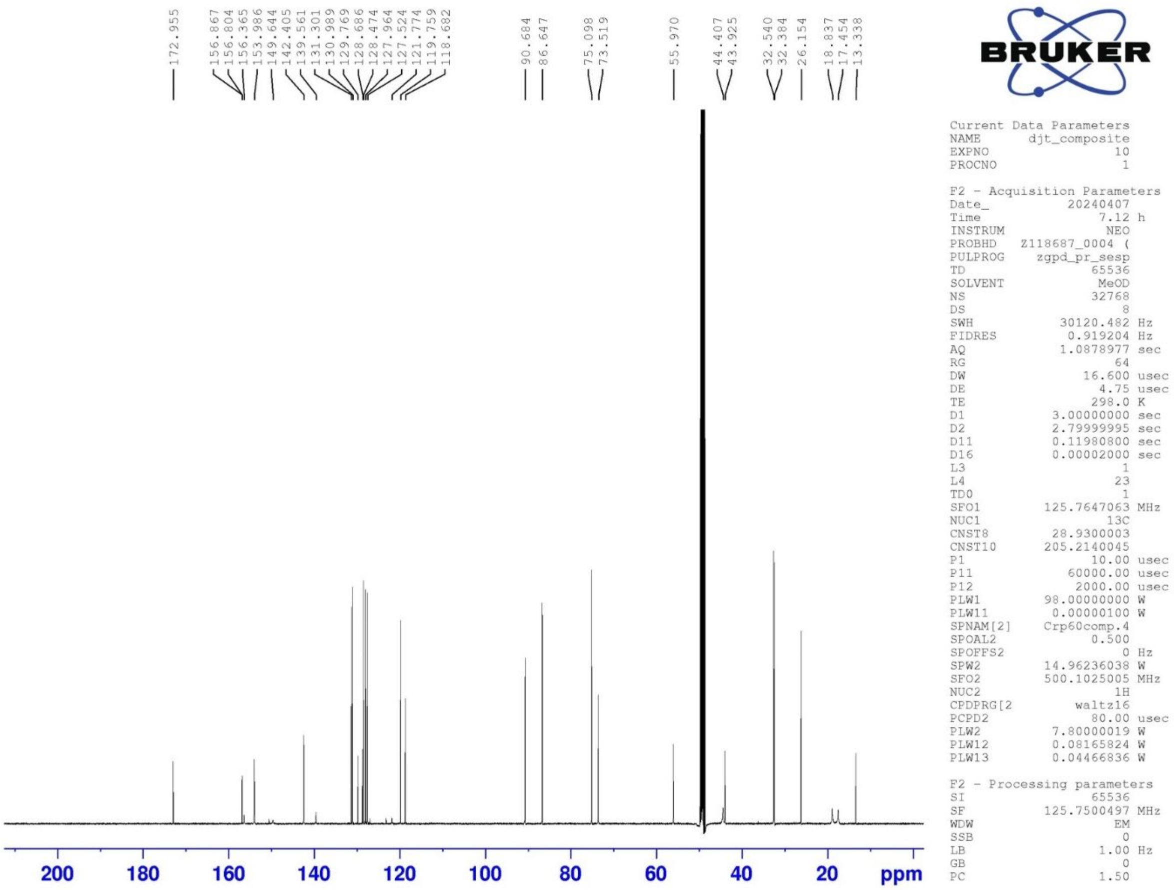

^{13}C NMR (125 MHz, MeOD)

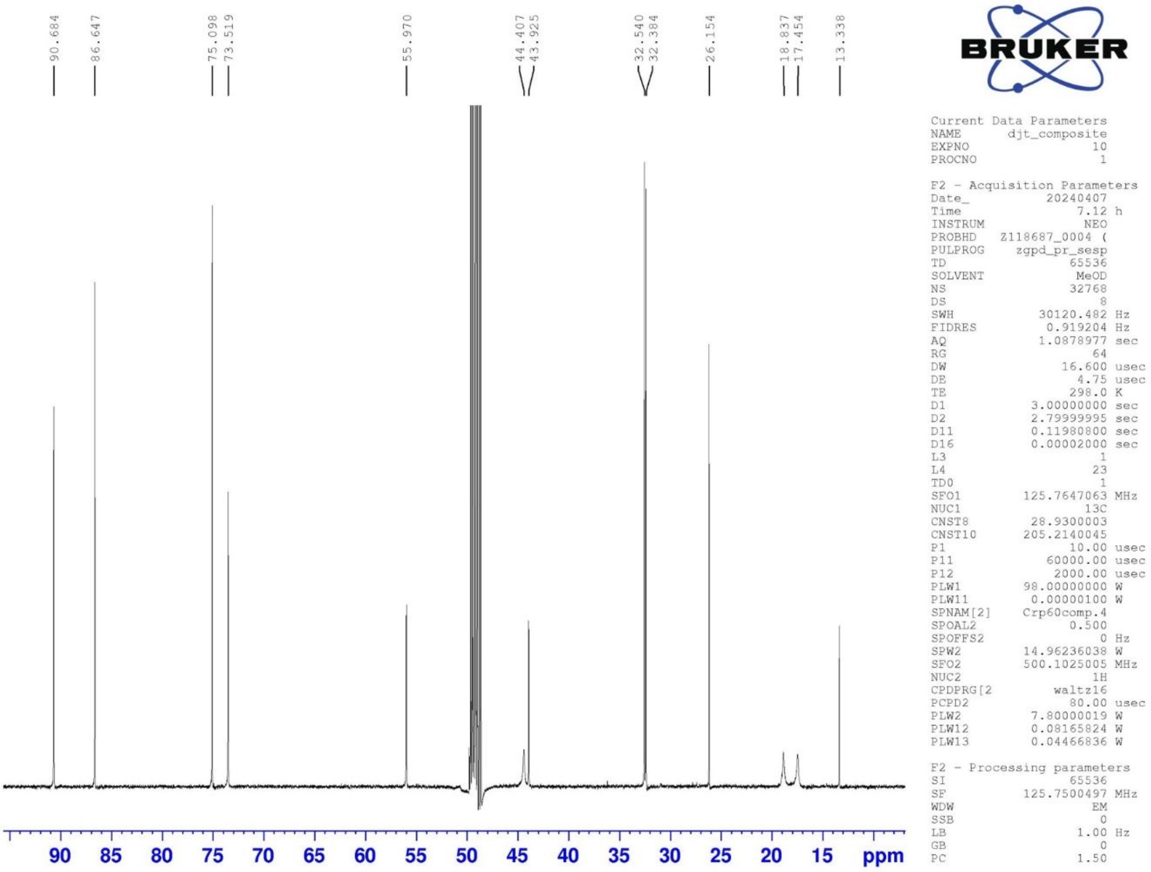

^{13}C NMR (125 MHz, MeOD) (expanded region)

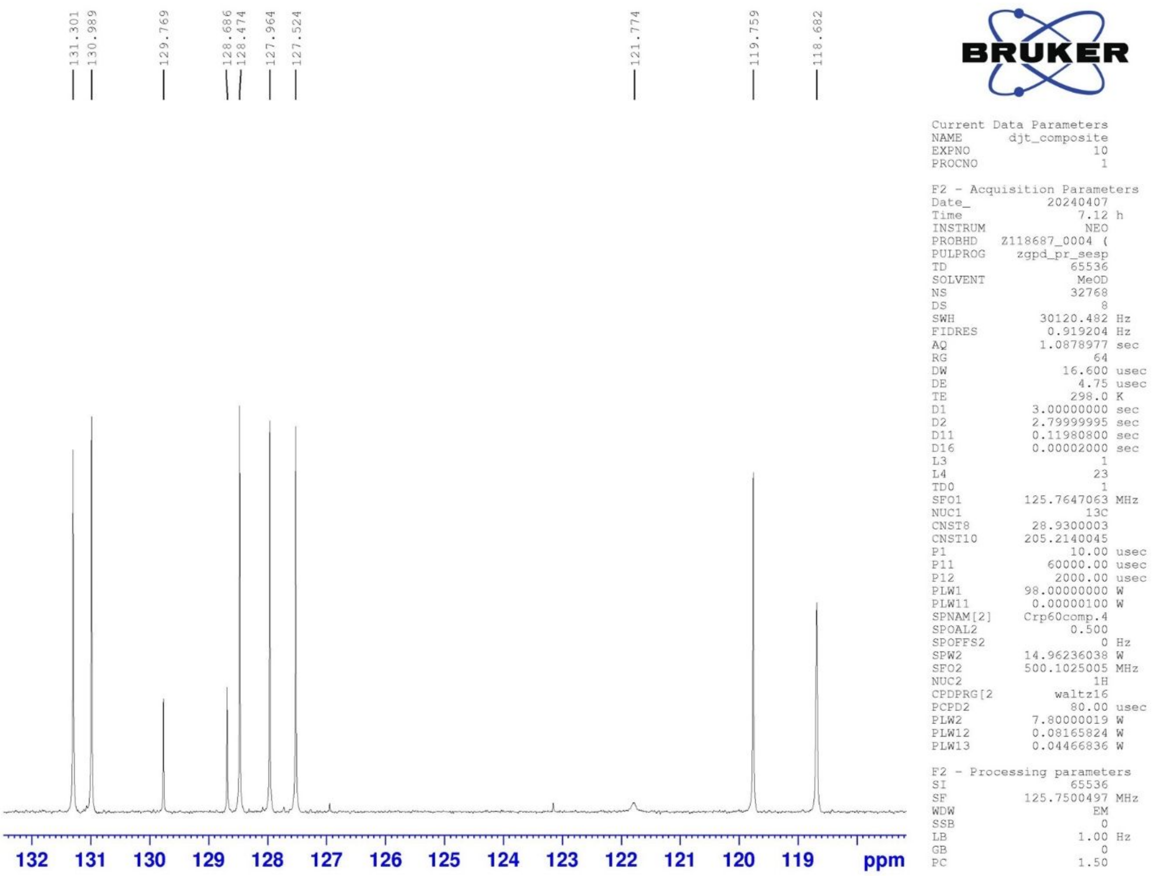

^{13}C NMR (125 MHz, MeOD) (expanded region)

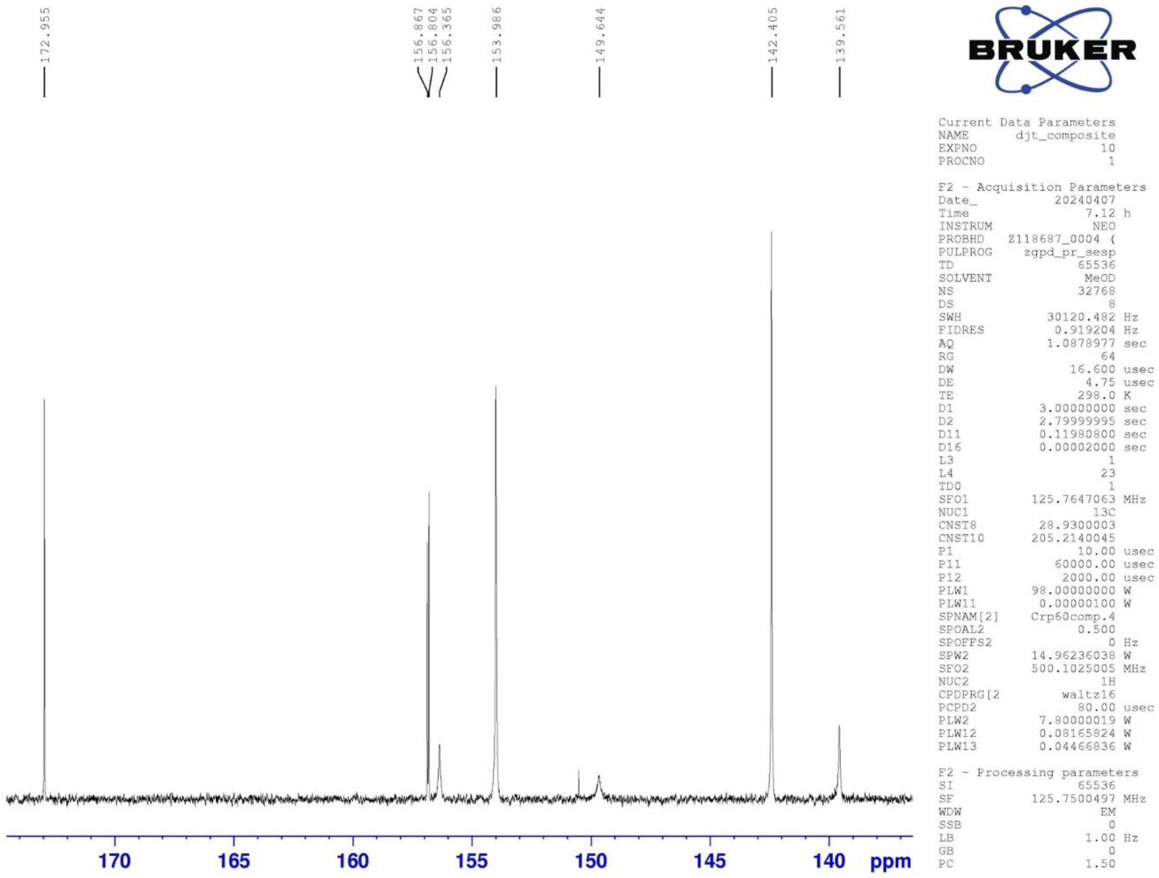

^{13}C NMR (125 MHz, MeOD) (expanded region)

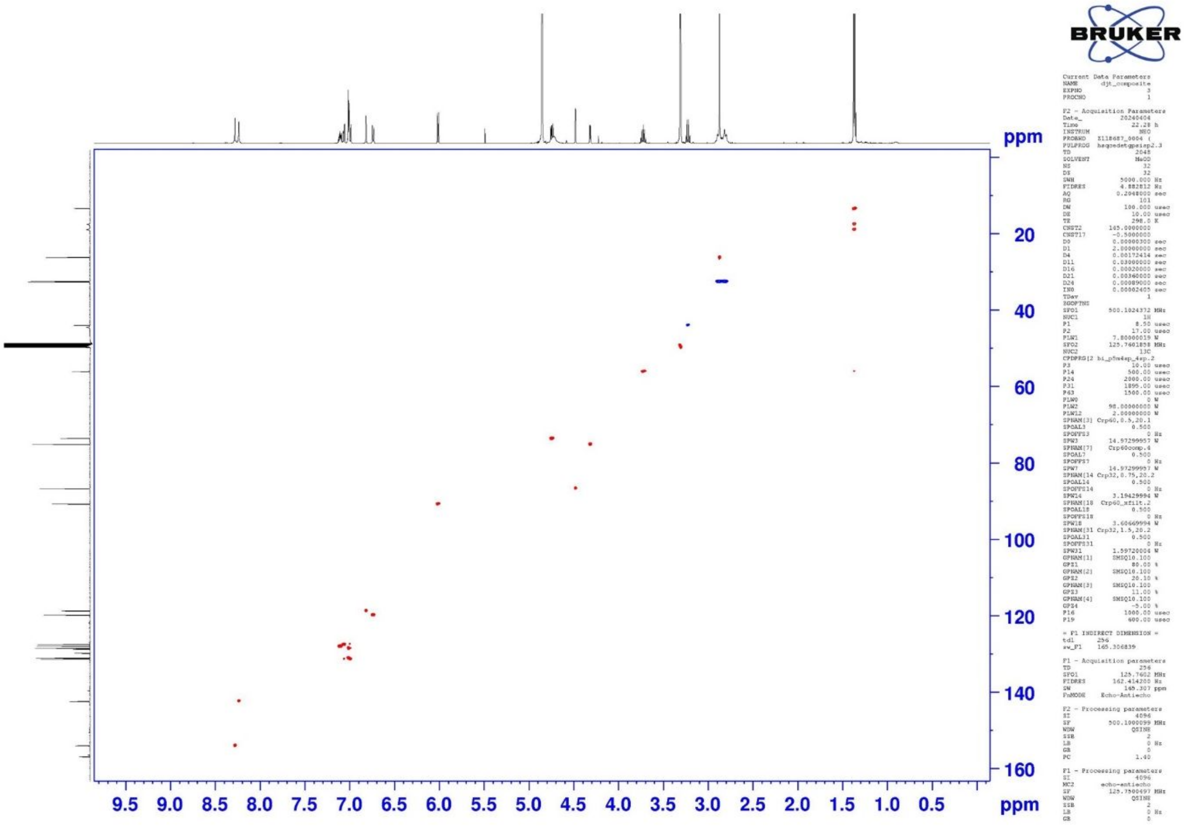

^{1}H-^{13}C HSQC (500 MHz, MeOD)

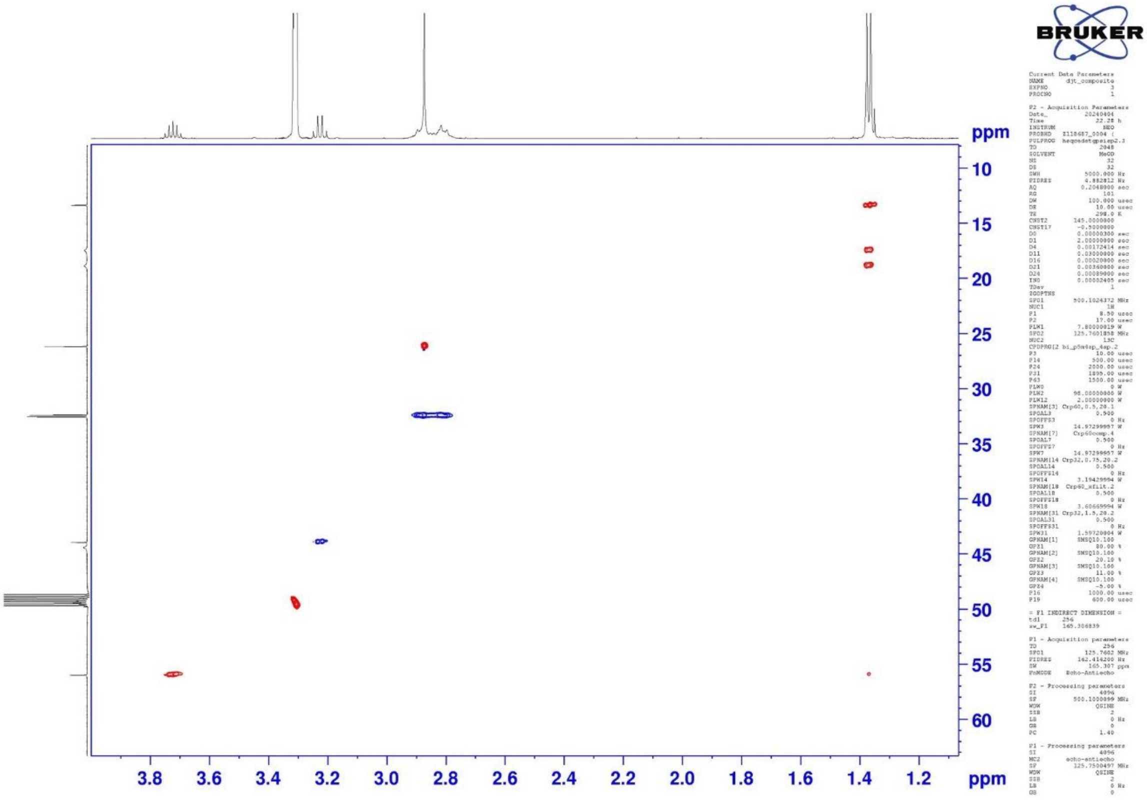

^{1}H-^{13}C HSQC (500 MHz, MeOD) (expanded region)

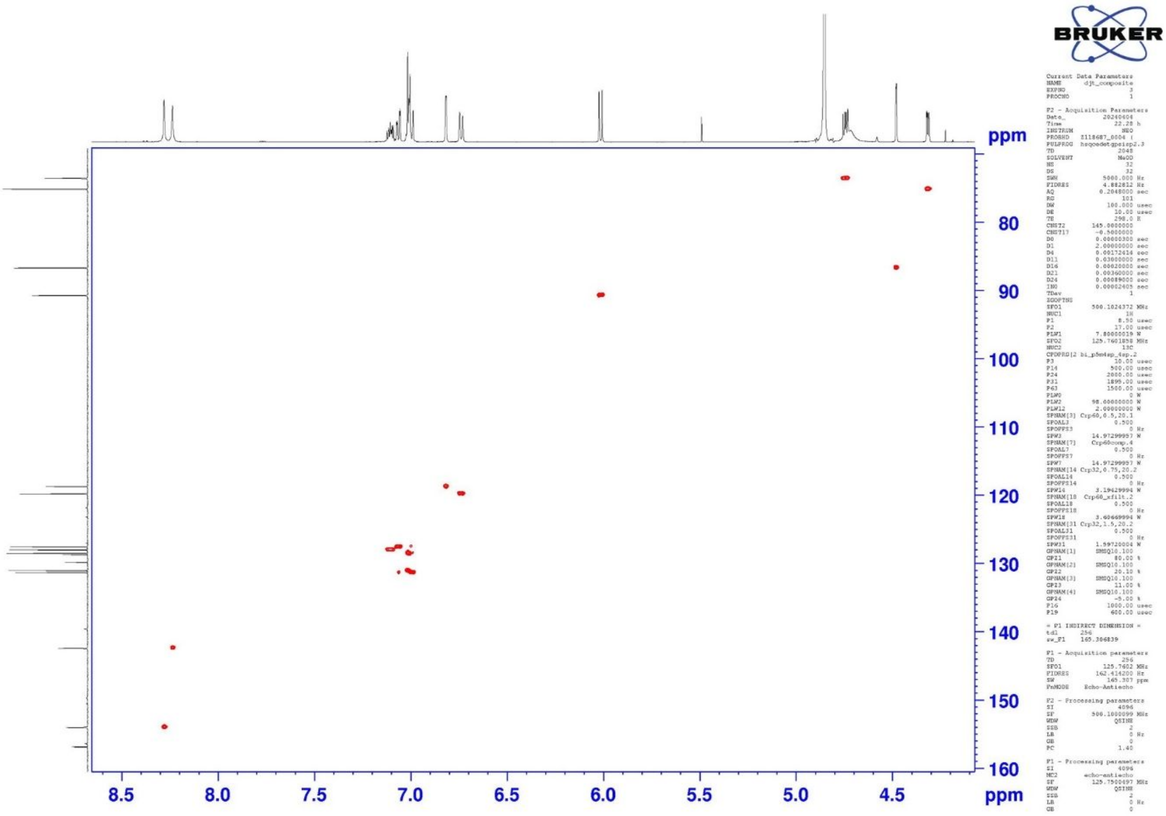

^{1}H-^{13}C HSQC (500 MHz, MeOD) (expanded region)

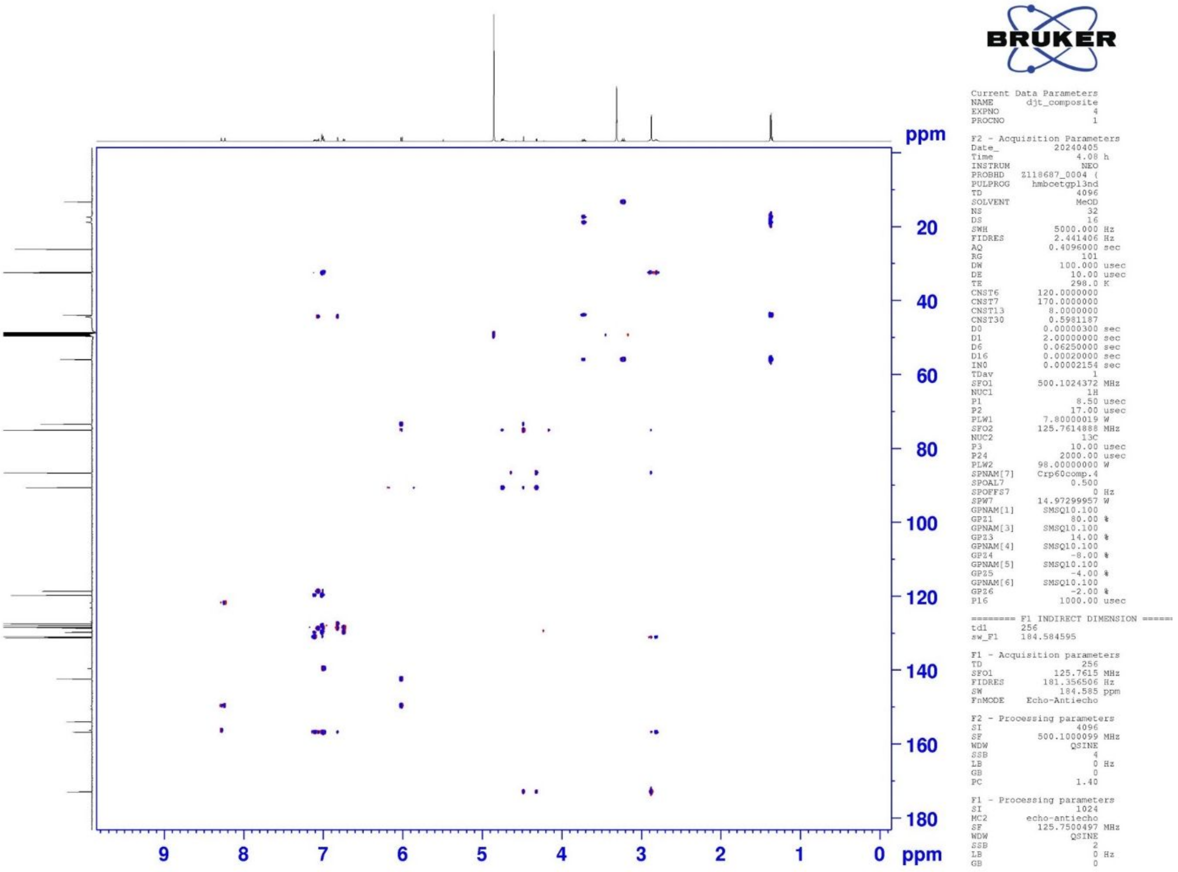

^{1}H-^{13}C HMBC (500 MHz, MeOD)

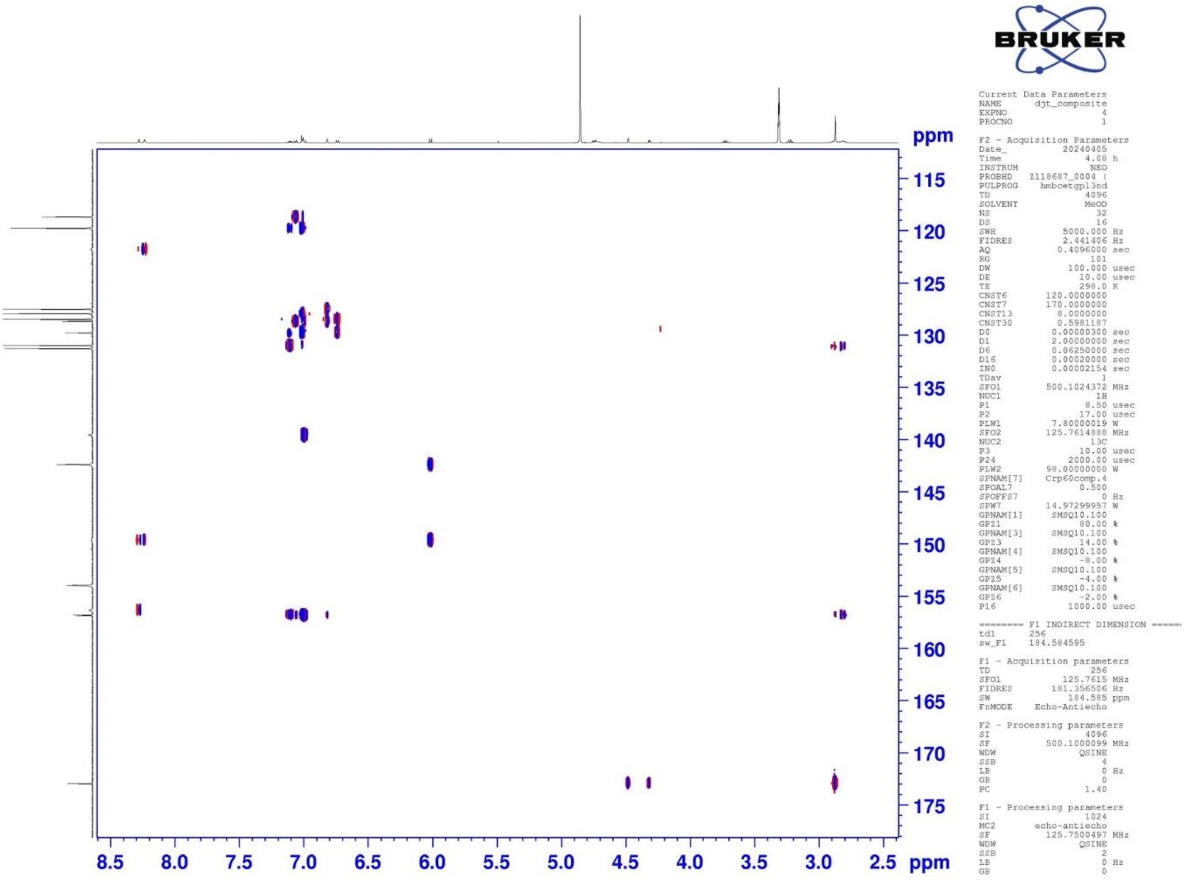

^{1}H-^{13}C HMBC (500 MHz, MeOD) (expanded region)

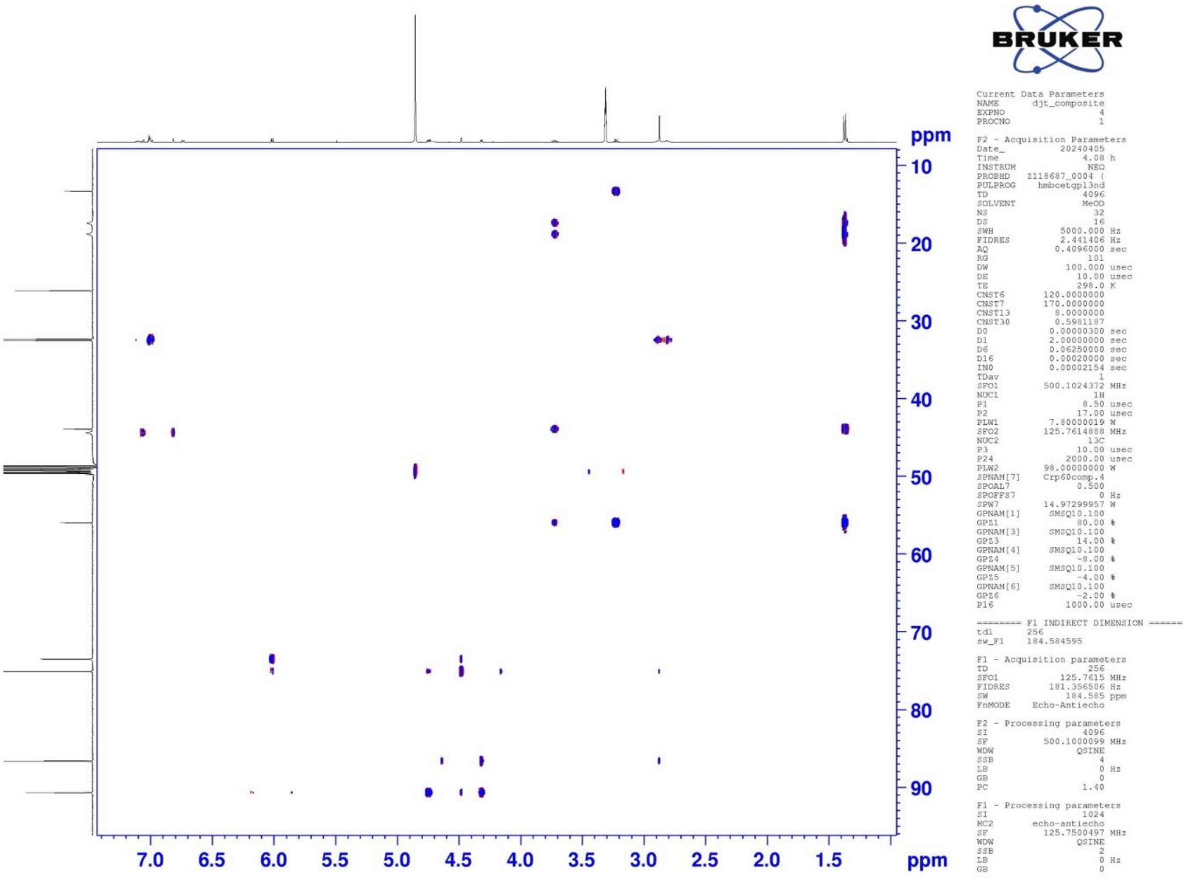

^{1}H-^{13}C HMBC (500 MHz, MeOD) (expanded region)

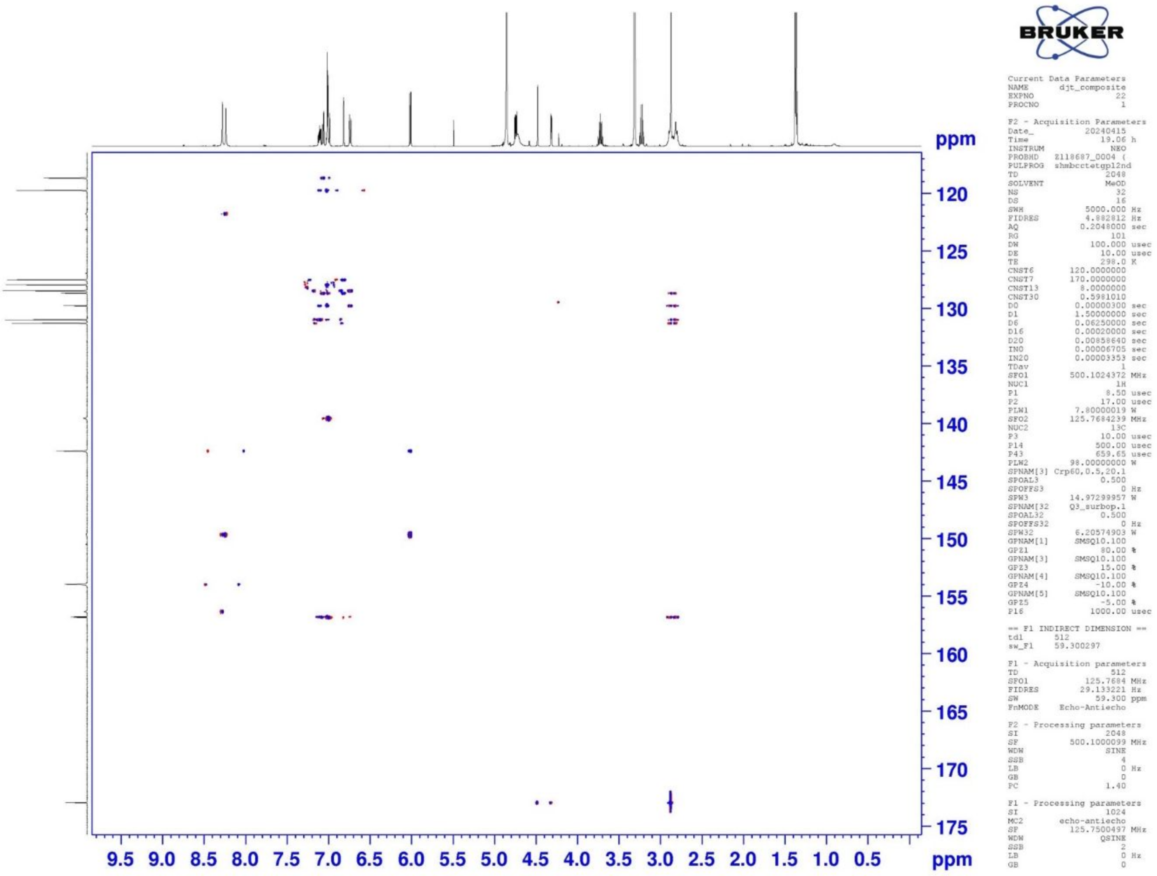

Selective ^{1}H-^{13}C HMBC (500 MHz, MeOD)

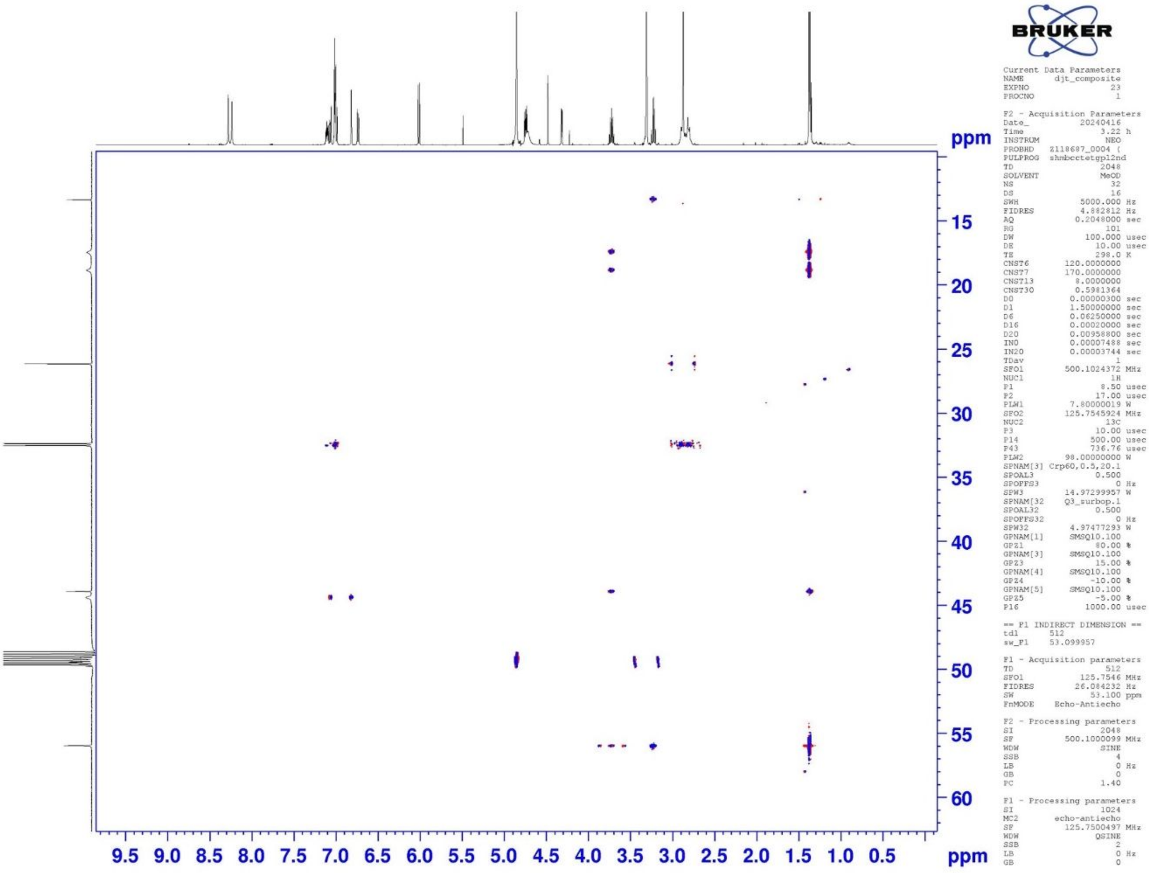

Selective ^{1}H-^{13}C HMBC (500 MHz, MeOD)

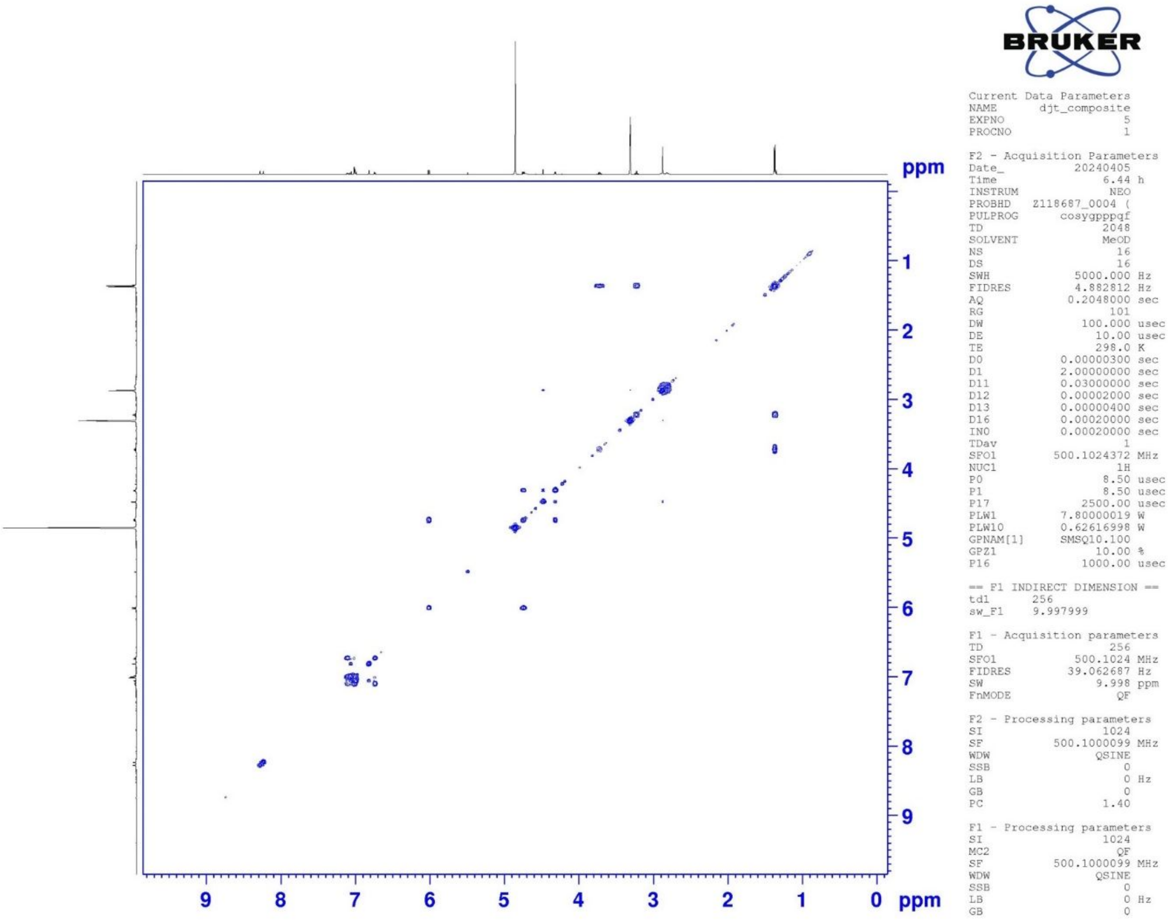

^{1}H-^{1}H COSY (500 MHz, MeOD)

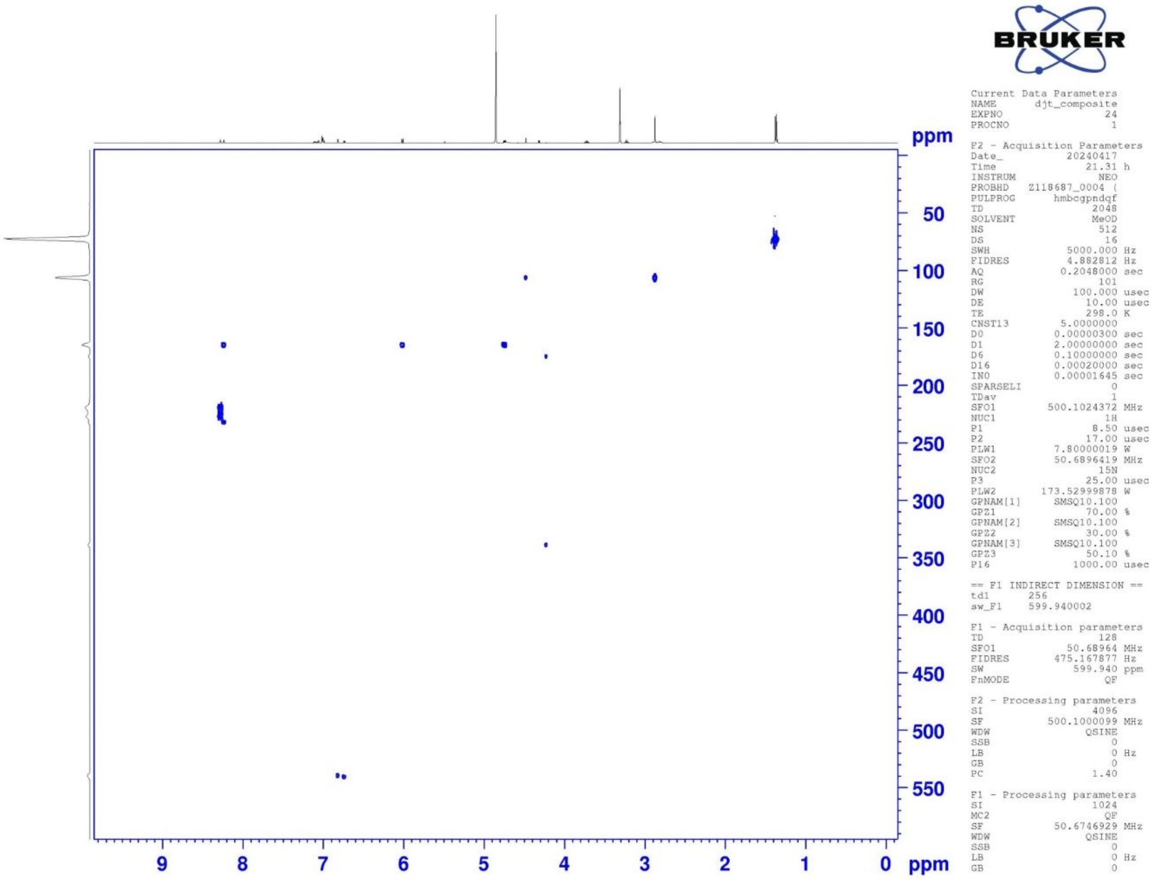

^{1}H-^{15}N HMBC (500 MHz, MeOD)

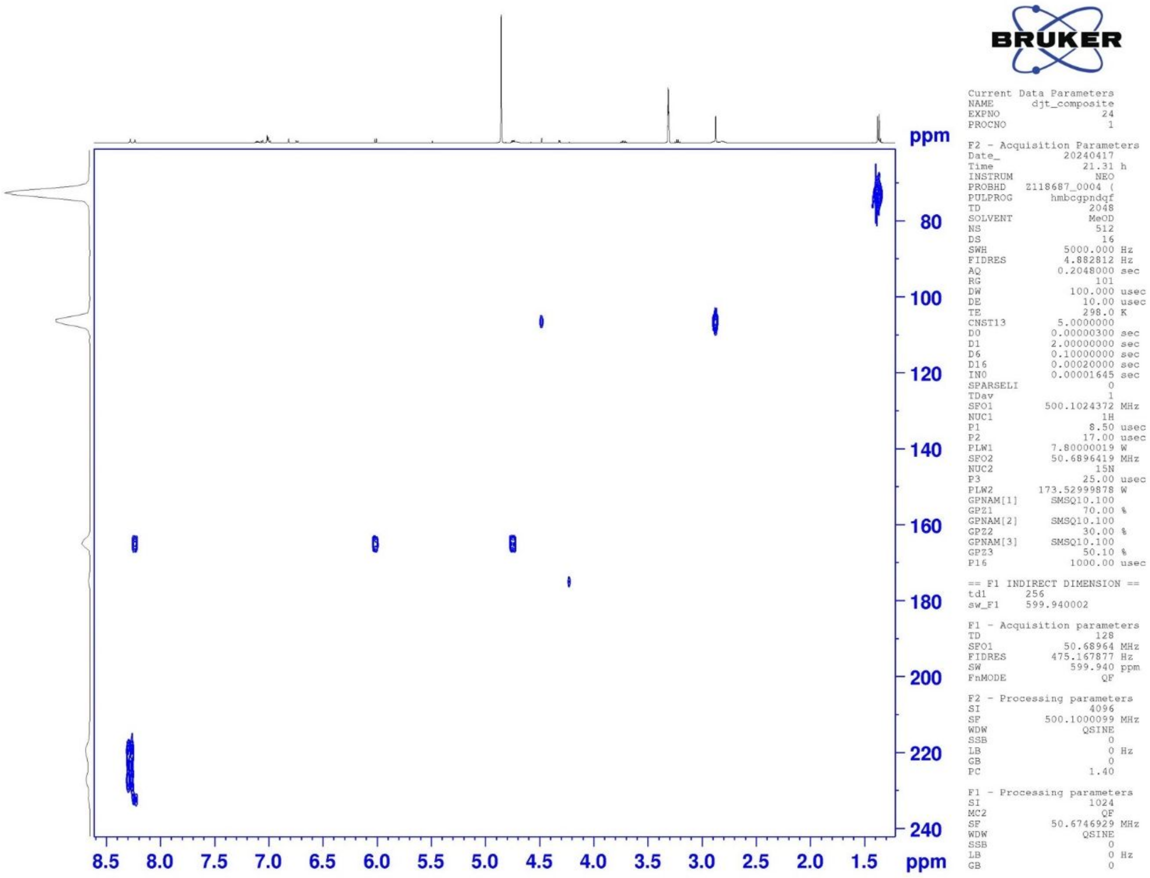

^{1}H-^{15}N HMBC (500 MHz, MeOD) (expanded region)

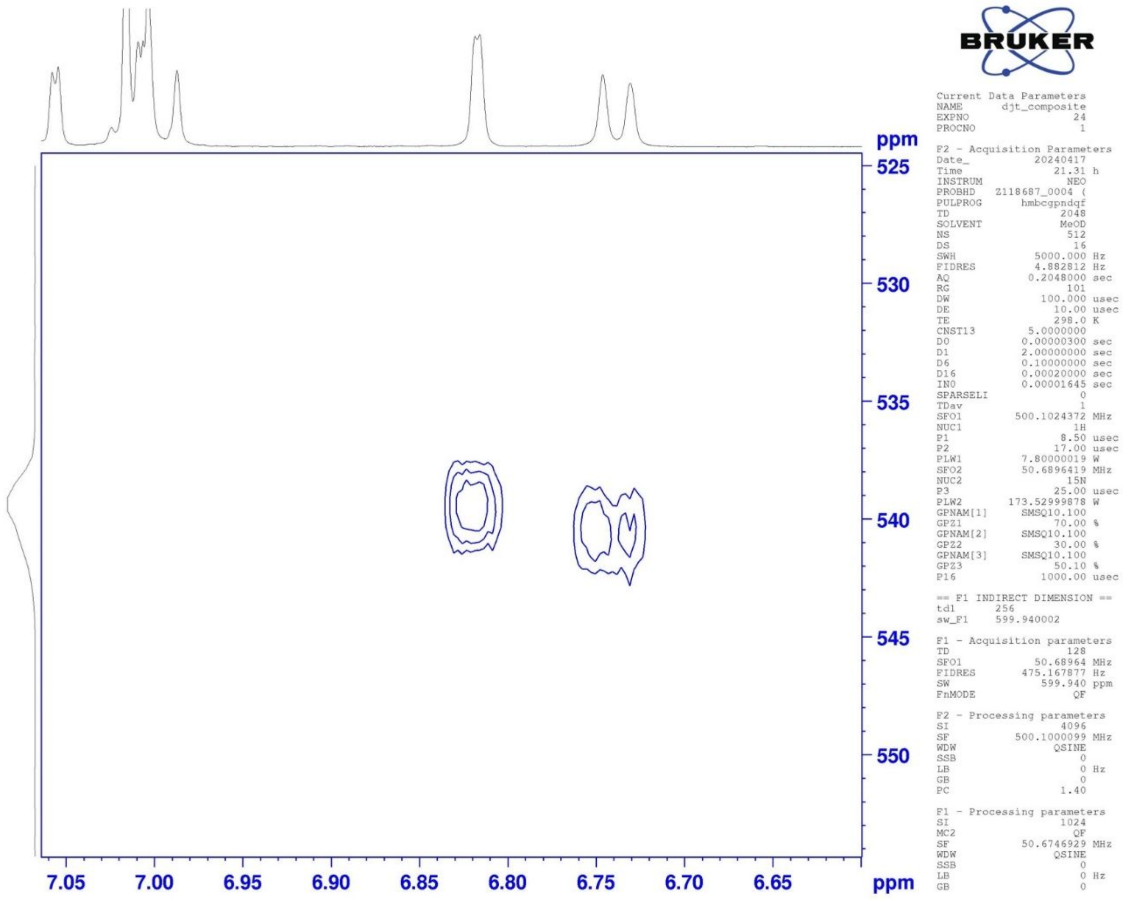

^{1}H-^{15}N HMBC (500 MHz, MeOD) (expanded region)

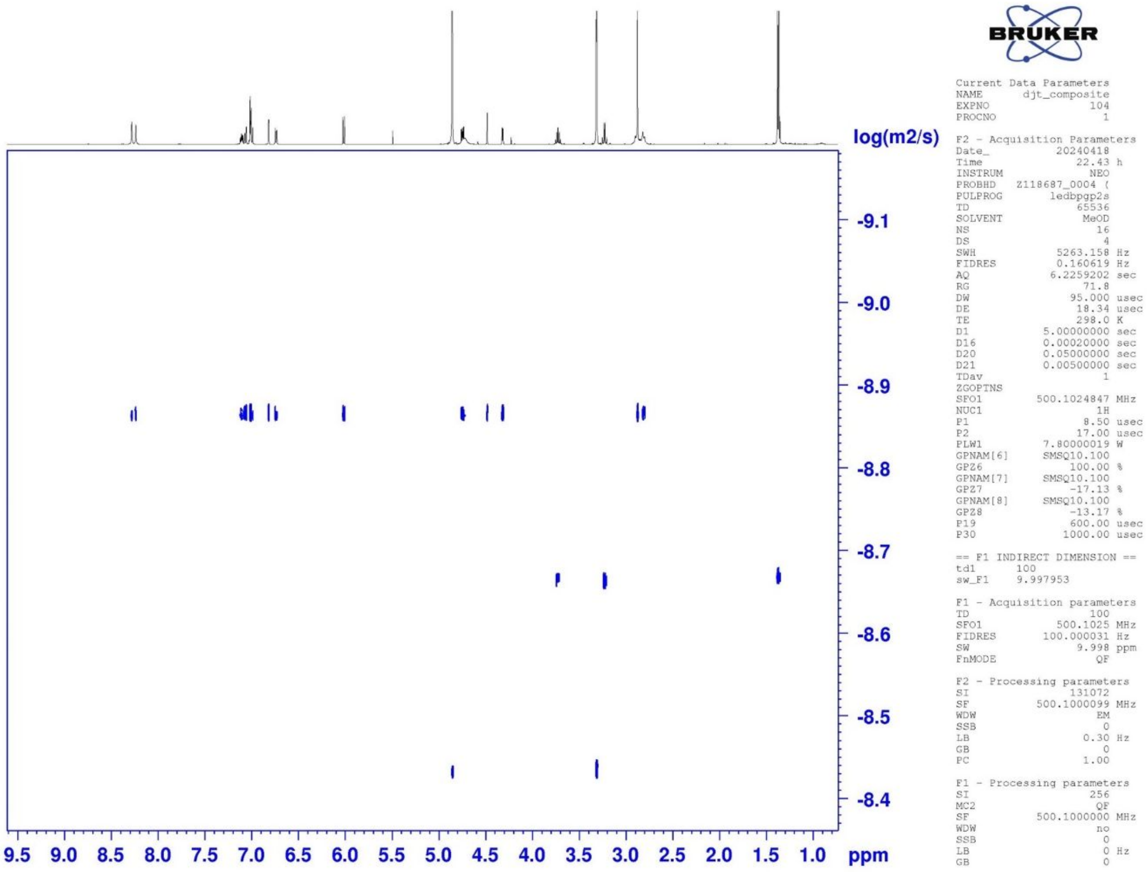

¹H DOSY (500 MHz, MeOD)

Table S1. Summary of NMR spectra

	δH	δC	δN	Assignment
Sugar unit:				
1′	6.01 (1H, d, $^{3}J_{HH}$ = 7.7 *Hz*)	90.68	-	CH
2′	4.74 (1H, dd, $^{3}J_{HH}$ = 7.7 *Hz*, $^{3}J_{HH}$ = 4.8 *Hz*)	73.52	-	CH
3′	4.32 (1H, dd, $^{3}J_{HH}$ = 4.8 *Hz*, $^{3}J_{HH}$ = 1.4 *Hz*)	75.10	-	CH
4′	4.48 (1H, d, $^{3}J_{HH}$ = 1.1 *Hz*)	86.65	-	CH
5′	-	172.96	-	C=O
6′	-	-	107	N
7′	2.87 (3H, s, overlapping)	26.15	-	CH_3
Adenine:				
1	-	-	219	NH
2	8.28 (1H, s)	130.99	-	CH
3	-	-	226	N
4	-	149.64 (br.)	-	quaternary
5	-	121.77 (br.)	-	quaternary
6	-	156.36 (br.)	-	quaternary
7	-	-	231	N
8	8.24 (1H, s)	142.40	-	CH
9	-	-	165	N
10				NH
Diazocine:				
11	4.72 (2H, br. s)	44.41 (br.)	-	CH_2
12	-	139.56 (br.)	-	quaternary
13	6.82 (1H, d, $^{4}J_{HH}$ = 1.3 *Hz*)	118.68	-	CH
14	-	156.87	-	quaternary
15	-	128.69	-	quaternary
16	7.00 (1H, d, $^{3}J_{HH}$ = 8.3 *Hz*)	131.30	-	CH
17	7.06 (1H, dd, $^{3}J_{HH}$ = 7.9 *Hz*, $^{4}J_{HH}$ = 1.7 *Hz*)	127.52	-	CH
18 and 19	2.78-2.92 (4H, multiplet, overlapping)	32.38 and 32.54	-	CH_2
20	-	129.77	-	quaternary
21	7.01 (1H, overlapping)	128.47	-	CH
22	7.01 (1H, overlapping)	130.99	-	CH
23	7.10 (1H, m)	127.96	-	CH
24	6.74 (1H, d, $^{3}J_{HH}$ = 7.8 *Hz*)	119.76	-	CH
25	-	156.80	-	quaternary
26	-	-	540	N=N
27	-	-	541	N=N

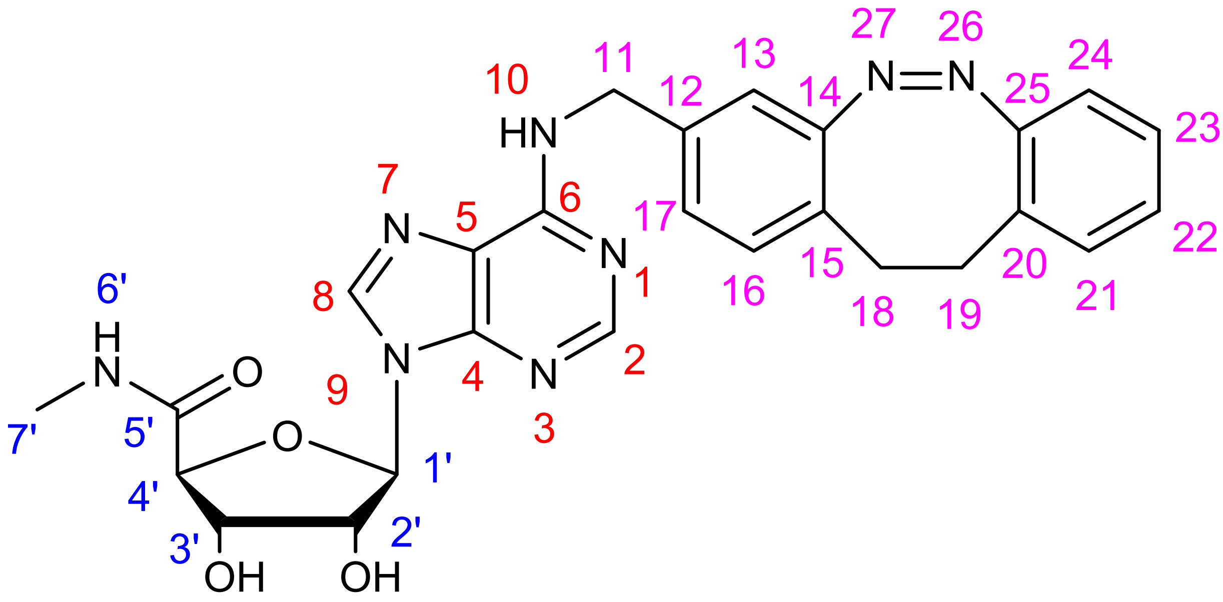

HN
N
N
N
N
O
O
N
H
OH
OH
N=N
1
2
3
4
5
6
7
8
9
10
11
12
13
14
15
16
17
18
19
20
21
22
23
24
25
26
27
1'
2'
3'
4'
5'
6'
7'

Mass spectral data of MRS7787 (4)

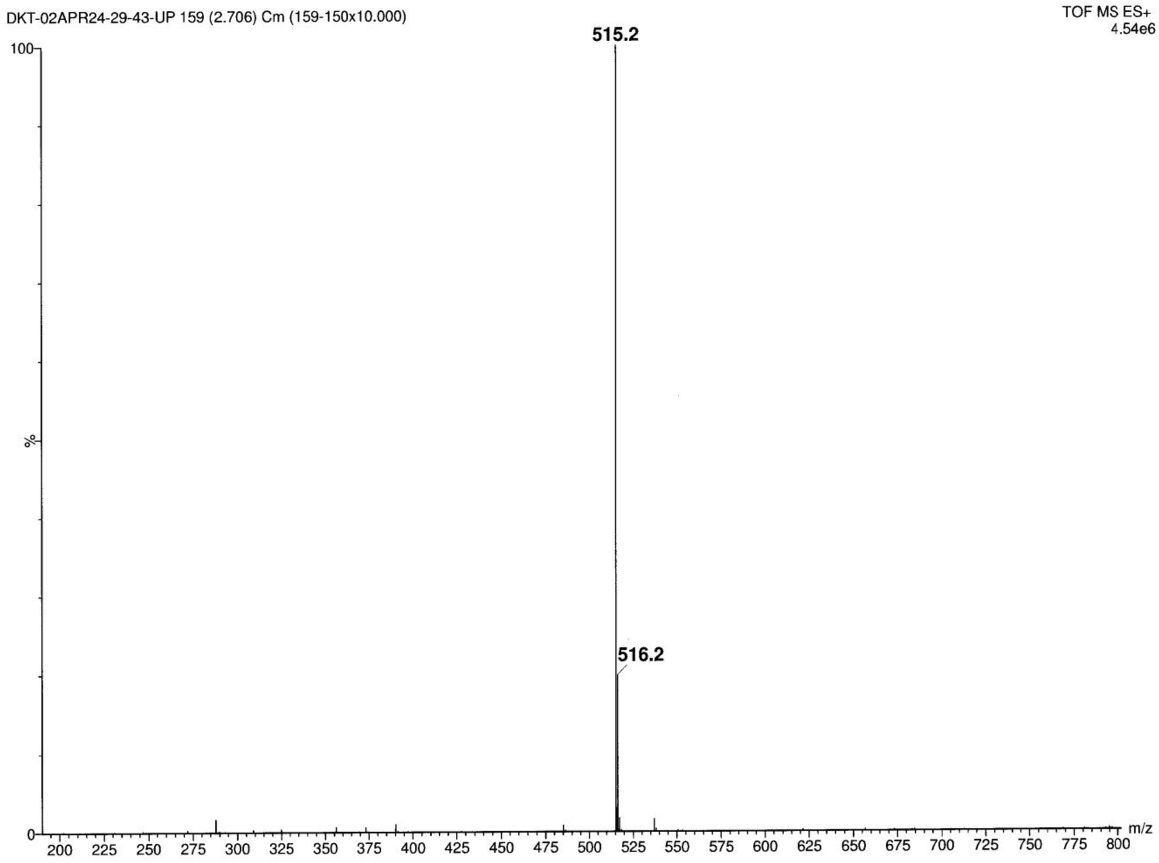

LRMS (FAB)

Elemental Composition Report **Page 1**

Single Mass Analysis
Tolerance = 5.0 mDa / DBE: min = -1.5, max = 100.0
Element prediction: Off
Number of isotope peaks used for i-FIT = 3

Monoisotopic Mass, Even Electron Ions
105 formula(e) evaluated with 1 results within limits (up to 50 closest results for each mass)
Elements Used:
C: 0-200 H: 0-200 N: 8-8 O: 0-30
DKT-02APR24-29-43-UP 166 (2.825) AM2 (Ar,25000.0,0.00,0.00); ABS
TOF MS ES+

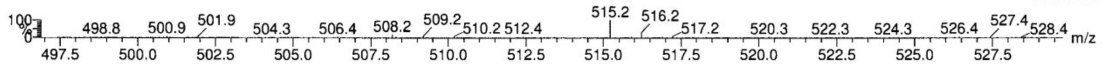

Minimum:				-1.5				
Maximum:		5.0	5.0	100.0				
Mass	Calc. Mass	mDa	PPM	DBE	i-FIT	Norm	Conf(%)	Formula
515.2155	515.2155	0.0	0.0	17.5	346.9	n/a	n/a	C26 H27 N8 O4

HRMS (API)

HPLC data of MRS7787 (4)

```
Data File C:\Chem32\1\DATA\TOSH\DKT-29-44000001.D
Sample Name: DKT-29-44

=====================================================================
Injection Date    : 9/23/2024 8:58:46 AM          Seq. Line :    1
Sample Name       : DKT-29-44                      Location : Vial 1
Acq. Operator     : TOSH                                Inj :    1
Acq. Instrument   : Instrument 1                 Inj Volume : 100 µl
Different Inj Volume from Sequence !      Actual Inj Volume : 10 µl
Sequence File     : C:\CHEM32\1\SEQUENCE\HARI.S
Method            : C:\CHEM32\1\METHODS\AN05100-20MIN.M
Last changed      : 8/7/2024 3:23:55 PM by HARI
```

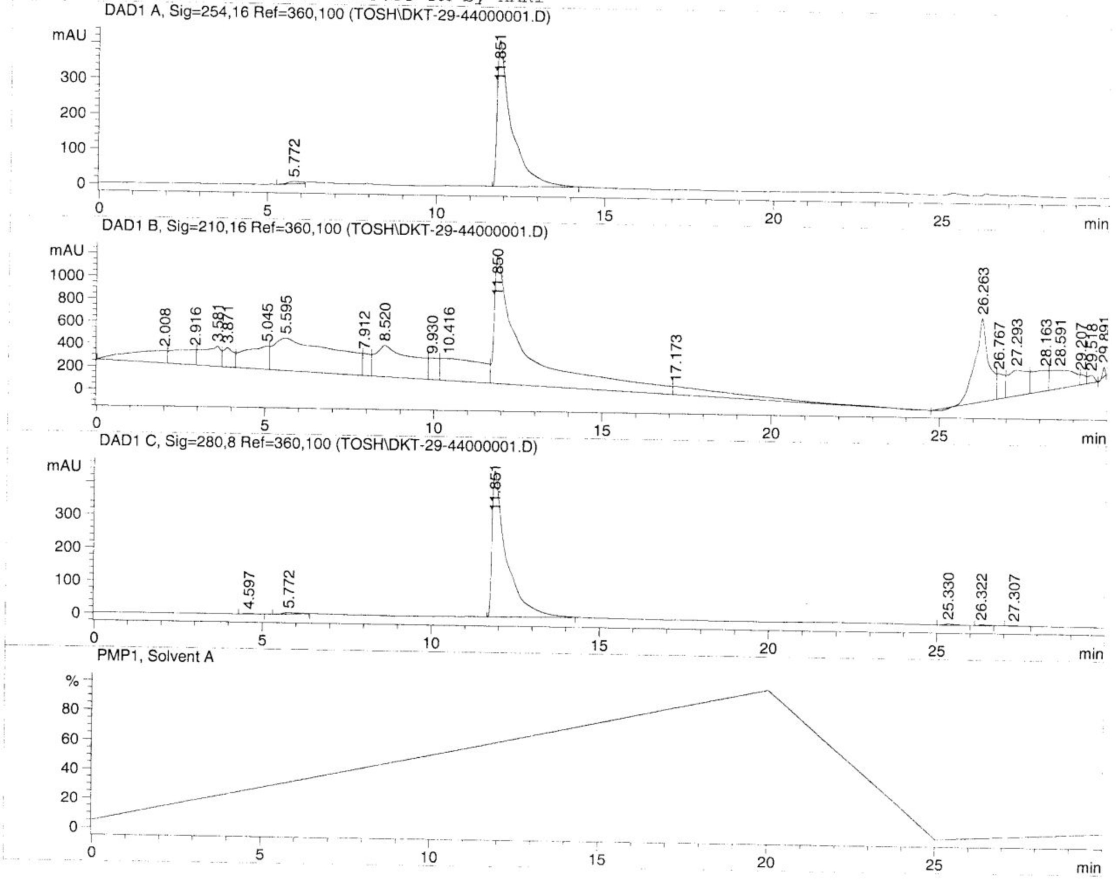


```
=====================================================================
                       Fraction Information
=====================================================================
Fraction collection off
=====================================================================
No Fractions found.
=====================================================================
```

Data File C:\Chem32\1\DATA\TOSH\DKT-29-44000001.D
Sample Name: DKT-29-44

===

Area Percent Report

===

Sorted By : Signal
Multiplier : 1.0000
Dilution : 1.0000
Use Multiplier & Dilution Factor with ISTDs

Signal 1: DAD1 A, Sig=254,16 Ref=360,100

Peak #	RetTime [min]	Type	Width [min]	Area [mAU*s]	Height [mAU]	Area %
1	5.772	BV	0.3749	185.70546	6.62692	1.4855
2	11.851	BB	0.4141	1.23158e4	412.71991	98.5145

Totals : 1.25015e4 419.34683

Signal 2: DAD1 B, Sig=210,16 Ref=360,100

Peak #	RetTime [min]	Type	Width [min]	Area [mAU*s]	Height [mAU]	Area %
1	2.008	BV	0.9592	8583.22559	107.07349	3.4794
2	2.916	VV	0.5627	6249.24756	132.39052	2.5332
3	3.581	VV	0.4631	6687.49170	179.32394	2.7109
4	3.871	VV	0.2764	3546.01953	169.27937	1.4374
5	5.045	VV	0.6280	1.01294e4	202.36102	4.1061
6	5.595	VV	1.5621	3.73275e4	284.98254	15.1314
7	7.912	VV	0.2125	3015.85742	190.72980	1.2225
8	8.520	VV	0.9613	2.11426e4	274.28650	8.5705
9	9.930	VV	0.2647	3959.05542	187.24802	1.6049
10	10.416	VV	1.0087	1.65239e4	193.03313	6.6983
11	11.850	VV	0.7744	6.95451e4	1135.68665	28.1914
12	17.173	VV	1.6463	1.01287e4	73.47244	4.1059
13	26.263	VV	0.3746	2.10851e4	735.08209	8.5472
14	26.767	VV	0.2197	3449.51660	221.46719	1.3983
15	27.293	VV	0.5325	9058.46777	222.70729	3.6720
16	28.163	VV	0.4154	6074.70996	177.43080	2.4625
17	28.591	VV	0.6002	7921.10791	157.64702	3.2110
18	29.207	VV	0.1612	959.98181	89.43039	0.3891
19	29.518	VV	0.2023	918.46283	67.70760	0.3723
20	29.891	VBA	0.0789	383.77750	81.55380	0.1556

Totals : 2.46689e5 4882.89362

Signal 3: DAD1 C, Sig=280,8 Ref=360,100

Peak #	RetTime [min]	Type	Width [min]	Area [mAU*s]	Height [mAU]	Area %
1	4.597	BB	0.2581	30.14443	1.41769	0.2226
2	5.772	BB	0.3863	123.22460	4.30008	0.9100
3	11.851	BB	0.4122	1.32113e4	442.66870	97.5592
4	25.330	BB	0.3304	94.91946	3.68629	0.7009
5	26.322	BB	0.2362	50.93788	3.06049	0.3762
6	27.307	BB	0.2981	31.29816	1.33228	0.2311

Totals : 1.35418e4 456.46553

IR spectrum of MRS7787 (4)

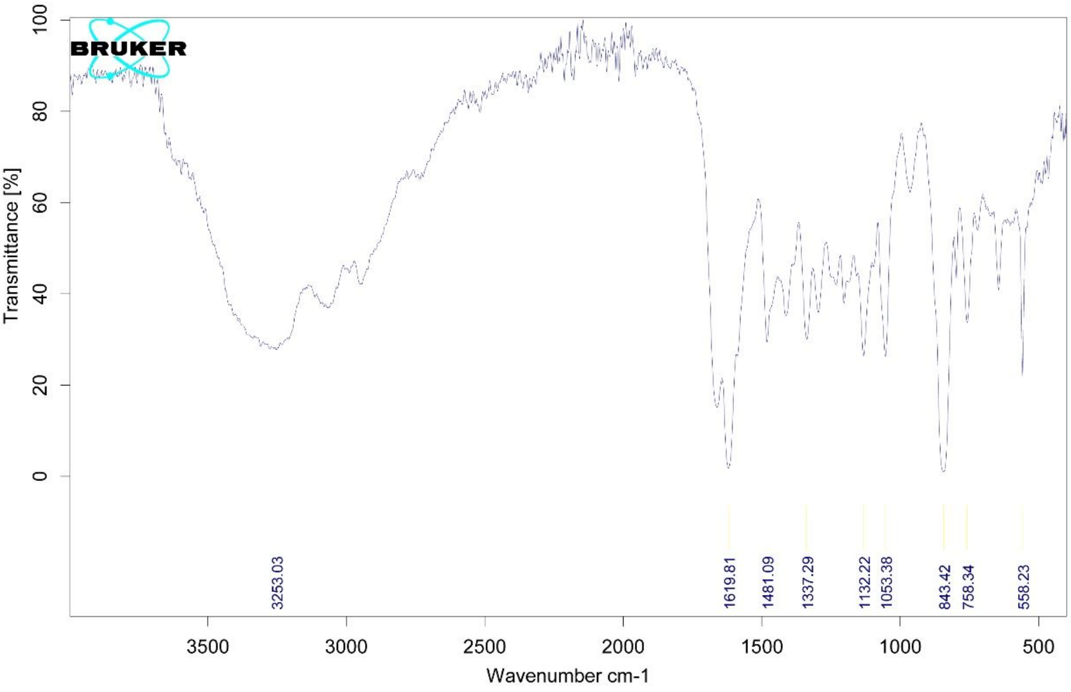

C:\Test\Test.5	Sample description	Instrument type and / or accessory	7/11/2024

Page 1/1

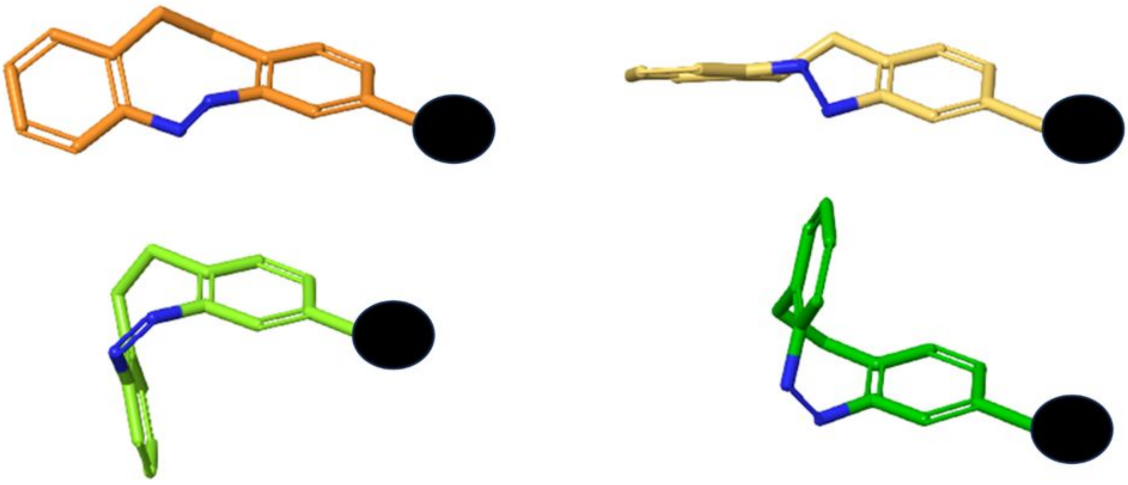

Figure S1. Different stereoisomers of the diazocene moiety. The *E* (orange and light orange) and *Z* (lime and green) stereoisomer forms are depicted. The black circles represent the common MRS7787 nucleosidic-like scaffold. The structures, showing the initial states of the two configurations, were made using Maestro graphical user interface (Schrödinger).

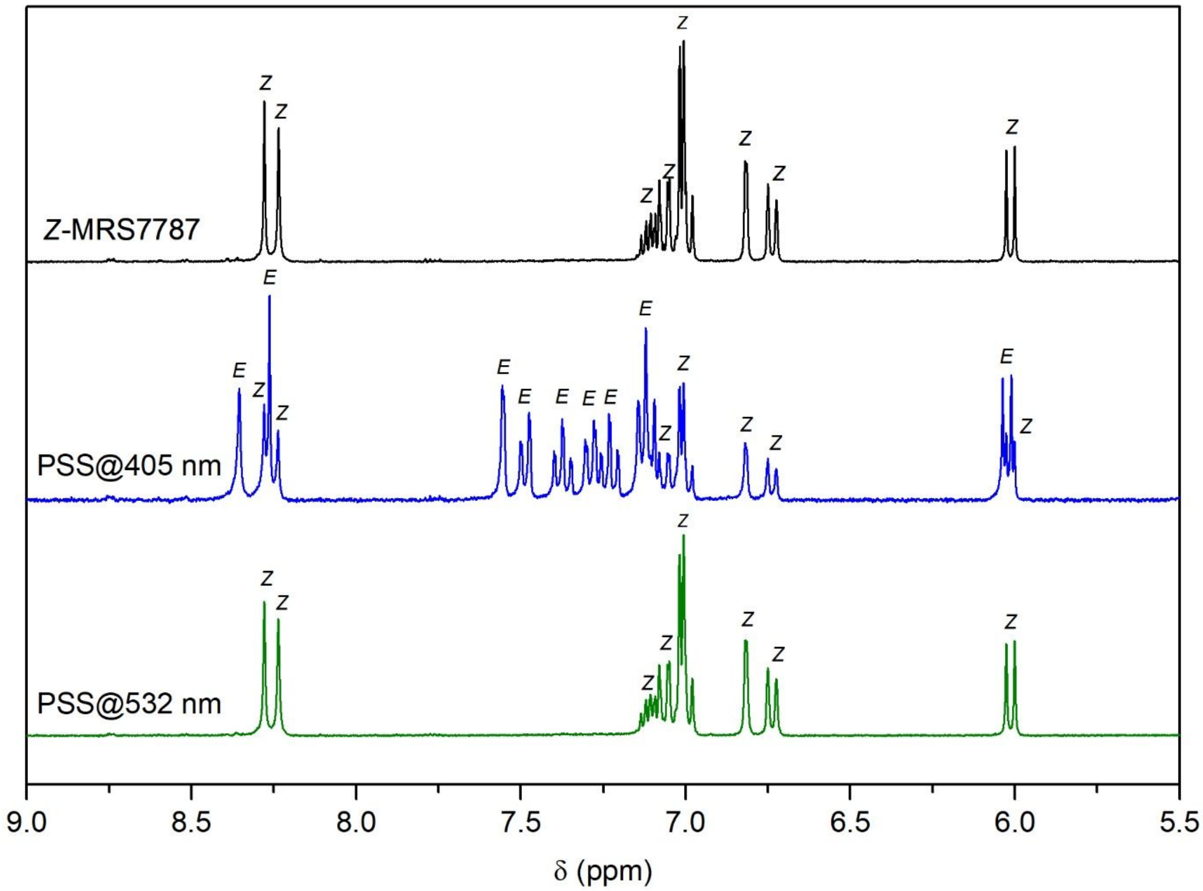

Figure S2. Photostationary mixtures of MRS7787 under irradiation. Low-field region of the ^{1}H NMR spectra (300 MHz, CD_3OD) of: the initial *Z*-MRS7787 compound in the dark (black, $c_{MRS7787}$ = 15 mM); the photostationary mixture obtained upon irradiation at λ_{exc} = 405 nm (t_{irr} = 25 min at 6.5 mW cm^{-2}) to promote *Z*-to-*E* photoisomerization (blue, $PSS_{Z\text{-}E}$@405 nm); the photostationary mixture obtained upon irradiation at λ_{exc} = 532 nm (t_{irr} = 80 min at 10.0 mW cm^{-2}) to promote *E*-to-*Z* back-photoisomerization (green, PSS@532 nm). The signals in all the spectra are assigned to the *Z* and *E* isomers of MRS7787. From the integrals of these signals, it can be concluded that: (1) PSS@405 nm is composed of 69% *E*-MRS7787 and 31% *Z*-MRS7787; (2) back-photoisomerization at λ_{exc} = 532 nm is quantitative and *E*-MRS7787 completely transforms to *Z*-MRS7787 under green light irradiation. From this data and UV-vis absorption spectra, the composition of PSS@405 nm in PBS:DMSO 98:2 could also be estimated: 61% *E*-MRS7787 and 39% *Z*-MRS7787.

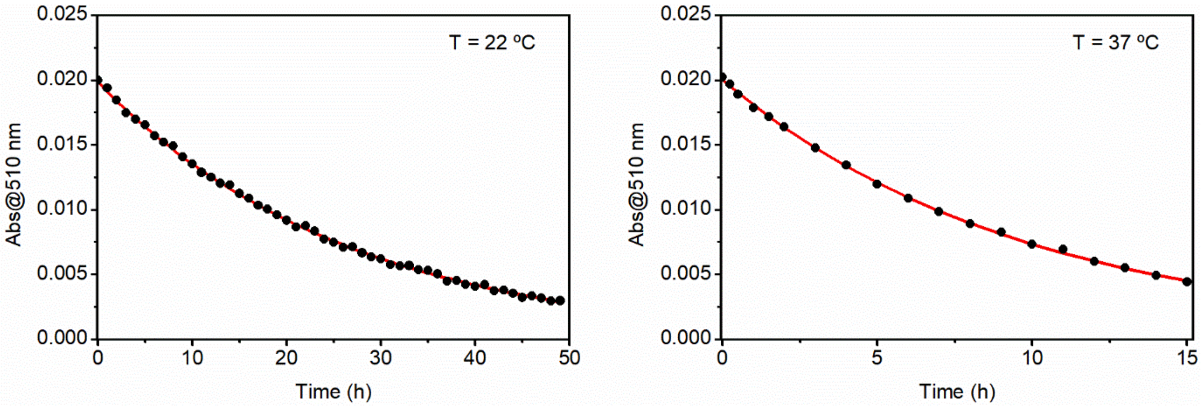

Figure S3. Effect of temperature on the half-life of *E*-MRS7787. Variation of the absorption at λ_{abs} = 510 nm for the *Z*-*E* photostationary state of MRS7787 in the dark at 22ºC (left panel) and 37ºC (right panel) in PBS:DMSO 98:2. At these conditions, thermal *E* → *Z* back-isomerization takes place, thus restoring the initial *Z* state of the compound. The experimental data (black circles) was adjusted to a monoexponential fit (red line), obtaining the following half-life times for *E*-MRS7787: $t_{1/2}$ = 18.4 h (22ºC) and 6.7 h (37ºC).

Biological Data

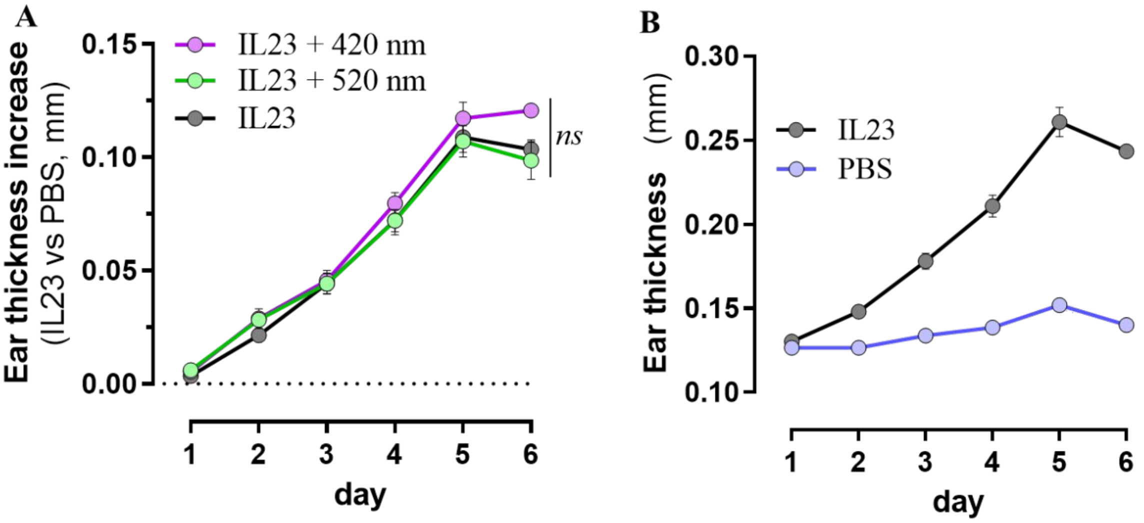

Figure S4. Effect of light on the IL-23-induced psoriatic phenotype. (**A**) Effect of light on IL23-induced ear thickness increase. Animals were treated with IL-23 or PBS (intradermal. i.d.) for four consecutive days (day 1, 2, 3 and 4). On days 3, 4 and 5 animals were administered intraperitoneally (i.p.) with vehicle (14.2% DMSO + 14.2% Tween80 in saline) before being irradiated with 405 nm or 520 nm light during 8 min. Ear thickness (mm) was measured upon anesthesia every single day during the 6-day duration of the protocol (*see* Figure S6). (**B**) Effect of vehicle (14.2% DMSO + 14.2% Tween80 in saline, i.p.) in the total ear thickness of both IL23 and PBS i.d. administered. Data is shown as mean ± S.E.M. (n = 5 mice per group). No significant (*ns*), one-way ANOVA with Dunnett's post-hoc test compared to IL23.

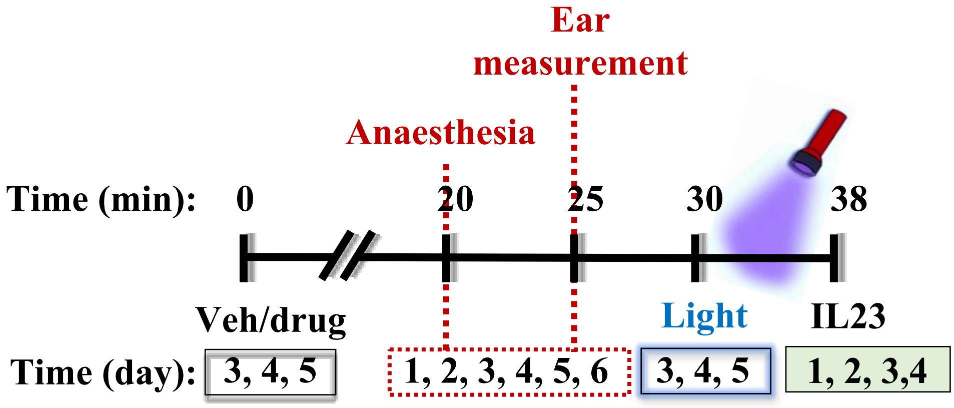

Figure S5. Temporal scheme for IL-23-induced mouse model of psoriasis. The ear thickness of animals was measured upon anaesthesia every single day for the 6-day duration of the protocol. Animals were treated with IL-23 (i.d.) in both ears during four consecutive days (day 1, 2, 3 and 4). At day 3, 4 and 5 animals were previously intraperitoneally (i.p.) administered with vehicle (Veh, 14.2% DMSO + 14.2% Tween80 in saline) and drugs (i.e., MRS5698 and MRS7787). When indicated the left animal's ear was irradiated with 420 or 520 nm light at day 3, 4 and 5.

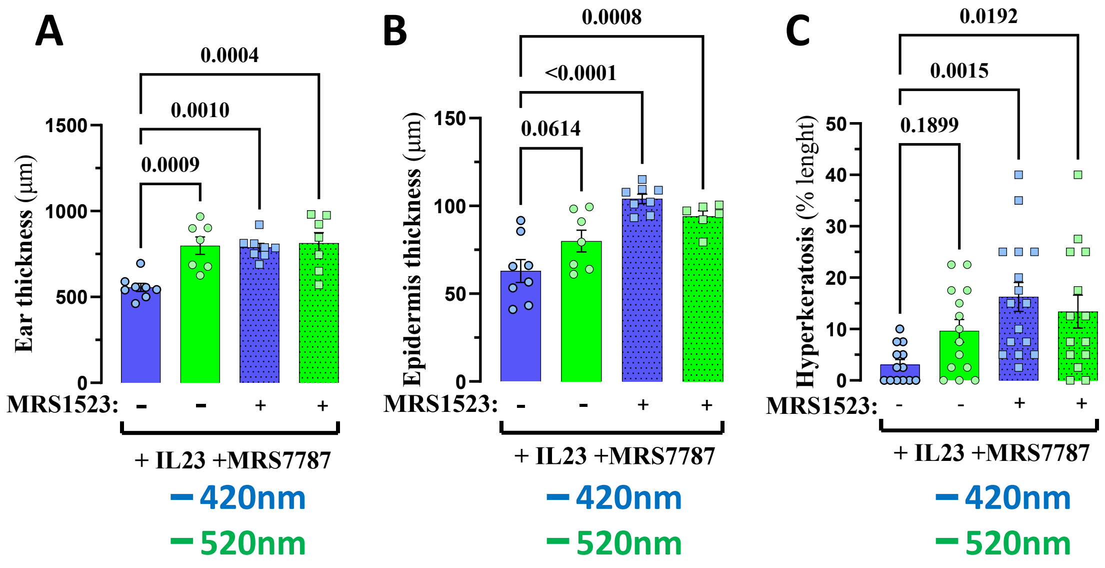

Figure S6. Quantification of *E*-MRS7787-mediated anti-inflammatory effects in the mouse psoriasis-like model. H&E-stained tissue sections were histologically evaluated for characteristic psoriasis markers, including total ear thickness, epidermal thickness, and hyperkeratosis in the ears of mice treated with MRS7787 plus MRS1523 (or vehicle). Measurements were taken on day 6 post-treatment. (A) Total ear thickness was assessed as an indicator of inflammation. (B) Epidermal thickness was measured to evaluate abnormal keratinocyte turnover and expansion. (C) Hyperkeratosis was quantified as a percentage of the affected skin length Data are represented as mean ± SEM, with 7–8 mice per group. Statistical analyses were performed using GraphPad Prism v10

References

(1) Siewertsen, R.; Neumann, H.; Buchheim-Stehn, B.; Herges, R.; Näther, C.; Renth, F.; Temps, F. Highly Efficient Reversible Z-E Photoisomerization of a Bridged Azobenzene with Visible Light through Resolved S(1)(n Pi*) Absorption Bands. *J Am Chem Soc* **2009**, *131* (43), 15594–15595. https://doi.org/10.1021/JA906547D.

(2) Sell, H.; Näther, C.; Herges, R. Amino-Substituted Diazocines as Pincer-Type Photochromic Switches. *Beilstein journal of organic chemistry* **2013**, *9*, 1–7. https://doi.org/10.3762/BJOC.9.1.

(3) Löw, R.; Rusch, T.; Röhricht, F.; Magnussen, O.; Herges, R. Diazocine-Functionalized TATA Platforms. *Beilstein journal of organic chemistry* **2019**, *15*, 1485–1490. https://doi.org/10.3762/BJOC.15.150.

(4) Gallo-Rodriguez, C.; Ji, X. duo; Melman, N.; Siegman, B. D.; Sanders, L. H.; Orlina, J.; Fischer, B.; Pu, Q.; Olah, M. E.; van Galen, P. J. M.; Stiles, G. L.; Jacobson, K. A. Structure-Activity Relationships of N6-Benzyladenosine-5'-Uronamides as A3-Selective Adenosine Agonists. *J Med Chem* **1994**, *37* (5), 636–646. https://doi.org/10.1021/JM00031A014.

(5) Tosh, D. K.; Salmaso, V.; Rao, H.; Bitant, A.; Fisher, C. L.; Lieberman, D. I.; Vorbrüggen, H.; Reitman, M. L.; Gavrilova, O.; Gao, Z. G.; Auchampach, J. A.; Jacobson, K. A. Truncated (N)-Methanocarba Nucleosides as Partial Agonists at Mouse and Human A3 Adenosine Receptors: Affinity Enhancement by N6-(2-Phenylethyl) Substitution. *J Med Chem* **2020**, *63* (8), 4334–4348. https://doi.org/10.1021/ACS.JMEDCHEM.0C00235.

(6) Xu, F.; Wu, H.; Katritch, V.; Han, G. W.; Jacobson, K. A.; Gao, Z. G.; Cherezov, V.; Stevens, R. C. Structure of an Agonist-Bound Human A2A Adenosine Receptor. *Science* **2011**, *332* (6027), 322–327. https://doi.org/10.1126/SCIENCE.1202793.

(7) Lebon, G.; Edwards, P. C.; Leslie, A. G. W.; Tate, C. G. Molecular Determinants of CGS21680 Binding to the Human Adenosine A2A Receptor. *Mol Pharmacol* **2015**, *87* (6), 907–915. https://doi.org/10.1124/MOL.114.097360/-/DC1.

(8) Glukhova, A.; Thal, D. M.; Nguyen, A. T.; Vecchio, E. A.; Jörg, M.; Scammells, P. J.; May, L. T.; Sexton, P. M.; Christopoulos, A. Structure of the Adenosine A1 Receptor Reveals the Basis for Subtype Selectivity. *Cell* **2017**, *168* (5), 867-877.e13. https://doi.org/10.1016/j.cell.2017.01.042.

(9) Madhavi Sastry, G.; Adzhigirey, M.; Day, T.; Annabhimoju, R.; Sherman, W. Protein and Ligand Preparation: Parameters, Protocols, and Influence on Virtual Screening Enrichments. *J Comput Aided Mol Des* **2013**, *27* (3), 221–234. https://doi.org/10.1007/S10822-013-9644-8.

(10) Schrödinger Release 2021–1: Maestro, Schrödinger, LLC. New York, NY 2021.

(11) Sherman, W.; Day, T.; Jacobson, M. P.; Friesner, R. A.; Farid, R. Novel Procedure for Modeling Ligand/Receptor Induced Fit Effects. *J Med Chem* **2006**, *49* (2), 534–553. https://doi.org/10.1021/JM050540C.

(12) Sherman, W.; Beard, H. S.; Farid, R. Use of an Induced Fit Receptor Structure in Virtual Screening. *Chem Biol Drug Des* **2006**, *67* (1), 83–84. https://doi.org/10.1111/J.1747-0285.2005.00327.X.

(13) Groom, C. R.; Bruno, I. J.; Lightfoot, M. P.; Ward, S. C. The Cambridge Structural Database. *Acta Crystallogr B Struct Sci Cryst Eng Mater* **2016**, *72* (Pt 2), 171–179. https://doi.org/10.1107/S2052520616003954.

(14) Friesner, R. A.; Banks, J. L.; Murphy, R. B.; Halgren, T. A.; Klicic, J. J.; Mainz, D. T.; Repasky, M. P.; Knoll, E. H.; Shelley, M.; Perry, J. K.; Shaw, D. E.; Francis, P.; Shenkin, P. S. Glide: A New Approach for Rapid, Accurate Docking and Scoring. 1. Method and Assessment of Docking Accuracy. *J Med Chem* **2004**, *47* (7), 1739–1749. https://doi.org/10.1021/jm0306430.
(15) Halgren, T. A.; Murphy, R. B.; Friesner, R. A.; Beard, H. S.; Frye, L. L.; Pollard, W. T.; Banks, J. L. Glide: A New Approach for Rapid, Accurate Docking and Scoring. 2. Enrichment Factors in Database Screening. *J Med Chem* **2004**, *47* (7), 1750–1759. https://doi.org/10.1021/JM030644S.
(16) Friesner, R. A.; Murphy, R. B.; Repasky, M. P.; Frye, L. L.; Greenwood, J. R.; Halgren, T. A.; Sanschagrin, P. C.; Mainz, D. T. Extra Precision Glide: Docking and Scoring Incorporating a Model of Hydrophobic Enclosure for Protein-Ligand Complexes. *J Med Chem* **2006**, *49* (21), 6177–6196. https://doi.org/10.1021/JM051256O.
(17) Longo, P. A.; Kavran, J. M.; Kim, M.-S.; Leahy, D. J. Transient Mammalian Cell Transfection with Polyethylenimine (PEI). *Methods Enzymol* **2013**, *529*, 227–240. https://doi.org/10.1016/B978-0-12-418687-3.00018-5.
(18) Taura, J.; Nolen, E. G.; Cabré, G.; Hernando, J.; Squarcialupi, L.; López-Cano, M.; Jacobson, K. A.; Fernández-Dueñas, V.; Ciruela, F. Remote Control of Movement Disorders Using a Photoactive Adenosine A 2A Receptor Antagonist. *Journal of Controlled Release* **2018**, *283*, 135–142. https://doi.org/10.1016/j.jconrel.2018.05.033.
(19) López-Cano, M.; Filgaira, I.; Nolen, E. G.; Cabré, G.; Hernando, J.; Tosh, D. K.; Jacobson, K. A.; Soler, C.; Ciruela, F. Optical Control of Adenosine A 3 Receptor Function in Psoriasis. *Pharmacol Res* **2021**, *170*, 105731. https://doi.org/10.1016/J.PHRS.2021.105731.
(20) Clark, J. D.; Gebhart, G. F.; Gonder, J. C.; Keeling, M. E.; Kohn, D. F. Special Report: The 1996 Guide for the Care and Use of Laboratory Animals. *ILAR journal / National Research Council, Institute of Laboratory Animal Resources* **1997**, *38* (1), 41–48.
(21) Manils, J.; Casas, E.; Viña-Vilaseca, A.; López-Cano, M.; Díez-Villanueva, A.; Gómez, D.; Marruecos, L.; Ferran, M.; Benito, C.; Perrino, F. W.; Vavouri, T.; de Anta, J. M.; Ciruela, F.; Soler, C. The Exonuclease Trex2 Shapes Psoriatic Phenotype. *Journal of Investigative Dermatology* **2016**, *136* (12), 2345–2355. https://doi.org/10.1016/j.jid.2016.05.122.
(22) Singh, T. P.; Zhang, H. H.; Hwang, S. T.; Farber, J. M. IL-23- and Imiquimod-Induced Models of Experimental Psoriasis in Mice. *Curr Protoc Immunol* **2019**, *125* (1). https://doi.org/10.1002/CPIM.71.
(23) Motulsky, H. J.; Brown, R. E. Detecting Outliers When Fitting Data with Nonlinear Regression - A New Method Based on Robust Nonlinear Regression and the False Discovery Rate. *BMC Bioinformatics* **2006**, *7*. https://doi.org/10.1186/1471-2105-7-123.
